# Lower lip inclination and chin prominence: interactive effects on facial aesthetics and implications for orthognathic treatment planning

**DOI:** 10.1186/s40902-026-00501-3

**Published:** 2026-04-06

**Authors:** Zhipeng Gui, Yueyang Hong, Lei Hou, Jiali Sun, Jialiang Huang, Mengmeng Lu

**Affiliations:** 1 Department of Oral and Maxillofacial Surgery, Shanghai Stomatological Hospital & School of Stomatology,Fudan University, Shanghai, 201102 People’s Republic of China; 2 Department of Pediatric Dentistry, Shanghai Stomatological Hospital & School of Stomatology, FudanUniversity, Shanghai, 201102 People’s Republic of China; 3 Department of Orthodontics, Shanghai Stomatological Hospital & School of Stomatology, Fudan University, Shanghai, 201102 People’s Republic of China

**Keywords:** Facial aesthetics, Lower lip inclination, Chin prominence, Interactive effects, Orthognathic surgery, Treatment planning, Genioplasty, Skeletal Class II, Chinese population

## Abstract

**Objective:**

To assess how lower lip inclination and chin prominence interact to influence facial profile aesthetics and to establish evidence-based angular criteria for orthognathic treatment planning.

**Methods:**

This study introduced a combined model using lower lip inclination angle (Li-Sbl) and lower lip-chin prominence angle (Lia-Pog′) to assess their interactive effects. Thirty standardized profile images combining three Li-Sbl angles (30°, 45°, 70°) with five Lia-Pog′ angles (0°–40°) were evaluated by 270 assessors (maxillofacial surgeons, orthodontists, skeletal Class II patients, laypeople) for aesthetic perceptions and surgical recommendations.

**Results:**

Gender-specific optimal configurations were identified: females achieved highest scores (7.81–8.19) at Li-Sbl 45°/Lia-Pog′ 20°; males at Li-Sbl 45°/Lia-Pog′ 10°–20° or Li-Sbl 70°/Lia-Pog′ 0° (6.31–7.60). Significant Li-Sbl × Lia-Pog′ interactions demonstrated compensation mechanisms. Critically, Li-Sbl ≤ 30° yielded consistently low ratings (< 5.0) regardless of chin positioning. Evaluator background significantly influenced perceptions and recommendations (*P* < 0.05).

**Conclusions:**

The Li-Sbl × Lia-Pog′ model provides the first quantitative framework for orthognathic treatment planning. It addresses two clinical decision points: identifying borderline cases requiring multidisciplinary consultation, and determining surgical approach where the Li-Sbl ≤ 30° threshold indicates isolated genioplasty is insufficient. We recommend gender-specific targets: Li-Sbl 45°/Lia-Pog′ 20° for females; Li-Sbl 45°/Lia-Pog′ 10°–20° or Li-Sbl 70°/Lia-Pog′ 0° for males. This framework enables evidence-based orthognathic surgical decision-making, reducing reliance on subjective assessment and improving treatment predictability.

**Supplementary Information:**

The online version contains supplementary material available at 10.1186/s40902-026-00501-3.

## Introduction

The lower facial profile strongly influences perceptions of facial attractiveness, gender characteristics, and age-related features [1]. The mentolabial sulcus, the soft tissue concavity between the lower lip and chin, plays a crucial role in maintaining facial harmony [2]. The morphology of this transitional zone depends on the coordinated interaction among mandibular position, lower incisor inclination, and soft tissue characteristics [3], making it a key factor in orthodontic and orthognathic surgical planning.

**Fig. 1:**
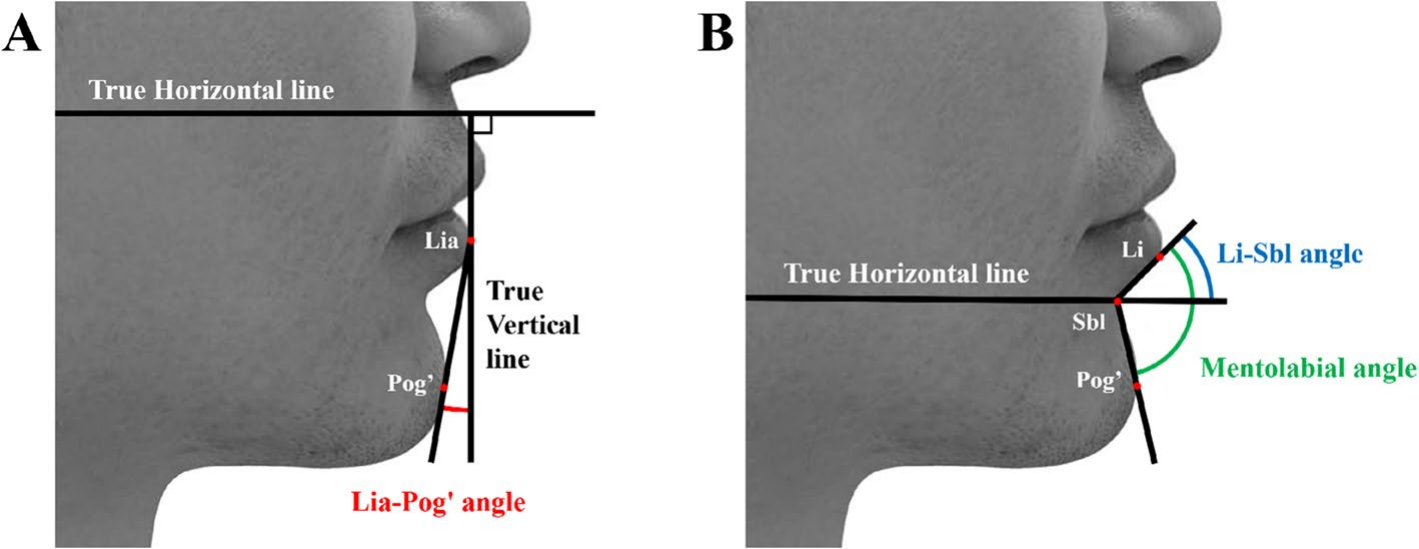
Two-parameter model for assessing the lower facial profile. **A** The lower lip-chin prominence angle (Lia-Pog' angle) indicates chin prominence relative to the lower lip. **B** The lower lip inclination angle (Li-Sbl angle) and the mentolabial angle. The Li-Sbl angle is defined as the angle formed by the horizontal plane and the long axis of the lower lip, representing the upper component of the mentolabial sulcus. Abbreviations: Lia, labrale inferius anterioris; Li, labrale inferius; Sbl, sublabiale; Pog', soft tissue pogonion

Advances in digital morphometric analysis have enabled precise quantification of lower facial structures. Recent studies have introduced several angular parameters for aesthetic evaluation, including the mentolabial angle, lower lip–chin prominence angle (Lia-Pog') (Fig. 1A), and facial contour angle [4–6]. Despite these methodological developments, most existing studies have assessed individual structural parameters in isolation rather than systematically examining how different facial components interact to shape overall aesthetic perception.

Current methods for assessing lower facial aesthetics have three major limitations. First, traditional techniques quantify lip morphology using protrusion measurements, typically expressed as the horizontal distance to reference lines such as the E-line or S-line [7, 8]. Although useful, these measurements cannot differentiate between aesthetic changes caused by anterior–posterior displacement of the entire lip and those arising from changes in lip inclination relative to the horizontal plane. Second, the inclination of the lower lip has not previously been evaluated systematically as an independent aesthetic parameter. The angle between the horizontal plane and the long axis of the lower lip, defined here as the lower lip inclination angle (Li-Sbl) (Fig. 1B), remains unexplored despite its potential significance for facial harmony. This parameter may be particularly important for skeletal Class II patients, whose lower lip inclination often compensates for mandibular retrusion [9]. Third, previous studies have separately analyzed lower lip and chin structures without investigating their interactive effects on aesthetic perception.

These knowledge gaps create two fundamental decision-making challenges for patients with lower facial aesthetic concerns. The first challenge is determining whether surgical intervention is necessary. This is particularly problematic in borderline cases where aesthetic deficiency is moderate rather than severe. Lower aesthetic scores do not automatically translate to treatment necessity, the threshold for surgical indication requires distinguishing subjective aesthetic perception from clinical treatment need [10]. Due to the multidisciplinary nature of these decisions, this often leads to difficulties or controversies in clinical decision-making. The second challenge is selecting the appropriate surgical approach between isolated genioplasty and comprehensive orthodontic-orthognathic surgery [11]. For instance, genioplasty may enhance chin projection but simultaneously deepen the mentolabial sulcus if lower lip position is inadequately considered [12]. Current practice lacks quantitative guidance for determining the selection threshold, potentially leading to suboptimal treatment outcomes.

Aesthetic perceptions differ systematically among evaluator groups. Evidence suggests that evaluators' own facial characteristics influence their aesthetic perception [4, 13]. Although previous research has documented preferences among clinicians and laypeople, skeletal Class II patients with mandibular hypoplasia remain underrepresented as evaluators. This population typically presents with chin retrusion and compromised mentolabial sulcus morphology [14]. While dentoalveolar compensation can achieve functional occlusion in some patients, residual aesthetic concerns frequently persist [15]. This requires them to navigate both challenges above, specifically whether surgical intervention is necessary and which approach is appropriate. Their perspectives, informed by direct experience with these clinical uncertainties, hold particular value for establishing treatment criteria.

To address these gaps, this study proposes a two-parameter model for evaluating lower facial profile aesthetics. We introduce the previously defined Li-Sbl angle alongside the established Lia-Pog' angle, enabling separate assessment of the upper component (lower lip posture) and lower component (chin prominence) of the mentolabial sulcus configuration. To our knowledge, this is the first study to systematically evaluate the Li-Sbl angle as an independent aesthetic parameter and examine its interactive effects with chin position.

The study aims are: (1) to identify optimal lower lip-chin configurations and quantify their interactive effects on aesthetic perception; (2) to compare aesthetic evaluations and surgical recommendations among maxillofacial surgeons, orthodontists, skeletal Class II patients, and laypeople; and (3) to establish quantitative decision thresholds for determining surgical necessity and selecting appropriate treatment approaches in young Chinese adults.

## Materials and methods

### Study design and ethical approval

This cross-sectional observational study was approved by the Ethics Committee of Shanghai Stomotological Hospital, Fudan University (ethics number [2023]020) and conducted following the Declaration of Helsinki. Written informed consent was obtained from both subjects (one male and one female). Evaluators were not required to provide individual consent, as approved by the Ethics Committee, since no sensitive personal data were collected, and all information remained anonymous to protect participant privacy.

### Standardized image acquisition

Standardized profile images were obtained from two Chinese volunteers: one female (22 years old) and one male (27 years old). Inclusion criteria were a skeletal Class I relationship, normal vertical facial proportions, complete dentition, facial symmetry, and no previous maxillofacial surgery or trauma.

Images were captured in a darkroom under standardized fluorescent lighting (5000 K color temperature) using a Nikon D850 camera equipped with a 105 mm macro lens. The camera was positioned at a fixed distance (1.5 m) from the subject, with constant settings (f/8 aperture, 1/125 s shutter speed, ISO 200). Subjects faced a suspended plumb line, which served as a calibration reference (True Vertical Line, TrV) and maintained a natural head position. To ensure relaxed perioral muscles, participants were instructed to produce a prolonged "M" sound before gently closing their lips. Right-side profile images were then captured.

### Image parameter construction

Using Naini’s soft-tissue measurement approach [16], four reference landmarks were identified using Adobe Illustrator: Sublabiale (Sbl), Labrale Inferius (Li), Labrale Inferius anterior (Lia), and soft-tissue Pogonion (Pog'). Image tracing was performed manually by the primary researcher and independently verified by three experienced researchers to ensure consistency, with all verifiers reaching unanimous agreement on landmark accuracy.

#### Lia-Pog' angle

A line parallel to the True Vertical Line (TrV) was drawn through Lia, and the angle formed by this line and the Lia-Pog' line was measured (Fig. 1A). Five angle increments (0°, 10°, 20°, 30°, and 40°) were established to simulate varying chin prominences.

#### Li-Sbl angle

A True Horizontal Line (TrH) was drawn through Sbl, and the angle formed between the Li-Sbl line and TrH was measured (Fig. 1B). Based on a pilot study (*n* = 30) demonstrating a normal distribution (50° ± 20°) within the target population, three angles were selected for clinical relevance: 30° (proclined lower lip), 45° (moderate inclination), and 70° (retroclined lower lip). These angles were sufficiently distinct for reliable visual assessment by evaluators.

**Fig. 2:**
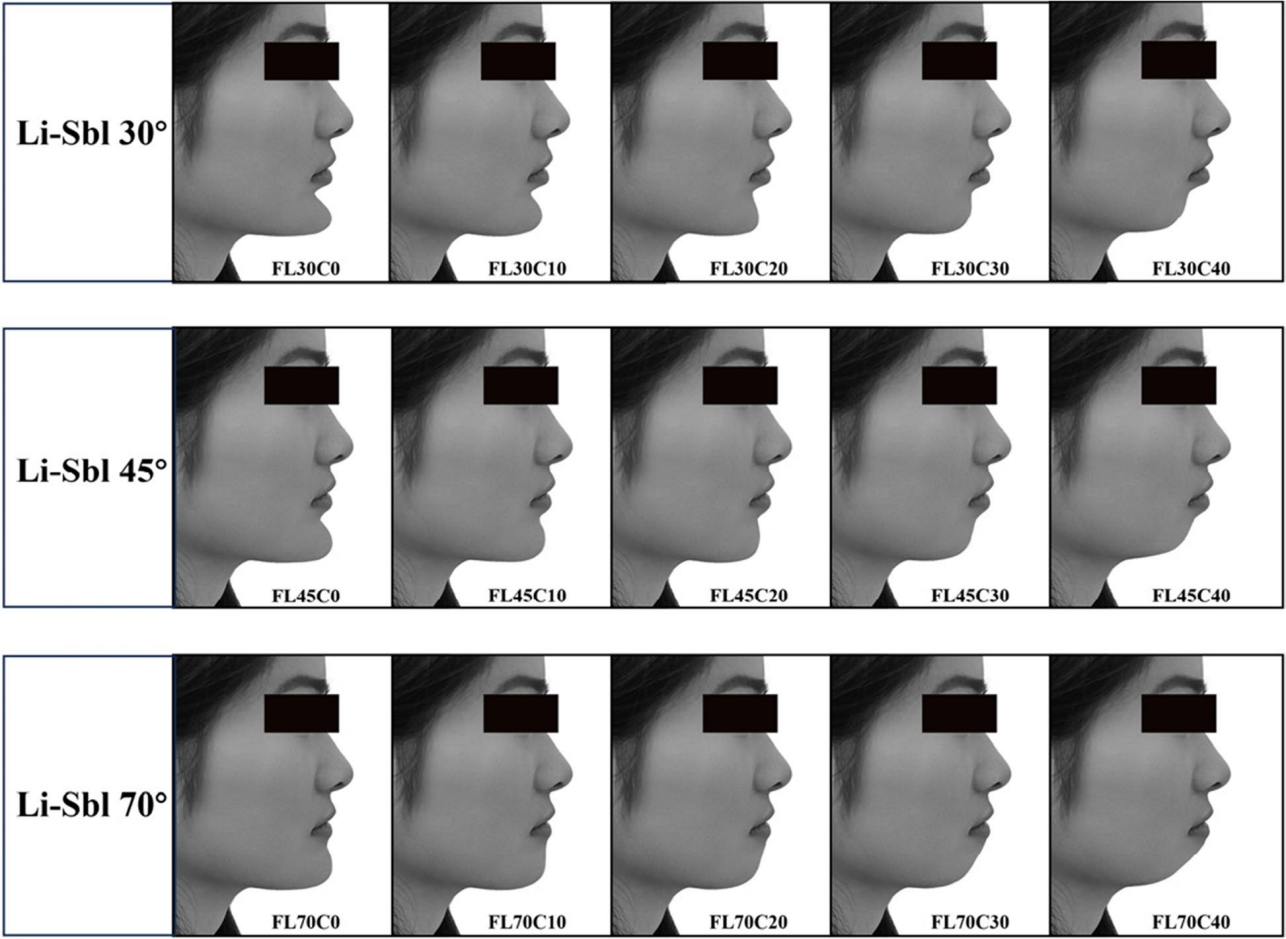
Female image configurations with varying Li-Sbl and Lia-Pog' angles. Fifteen standardized female images representing combinations of three Li-Sbl angles (30°, 45°, 70°) and five Lia-Pog' angles (0°, 10°, 20°, 30°, 40°). Image codes: FL30C0 to FL70C40 (F = female, L = Li-Sbl value, C = Lia-Pog' value)

**Fig. 3:**
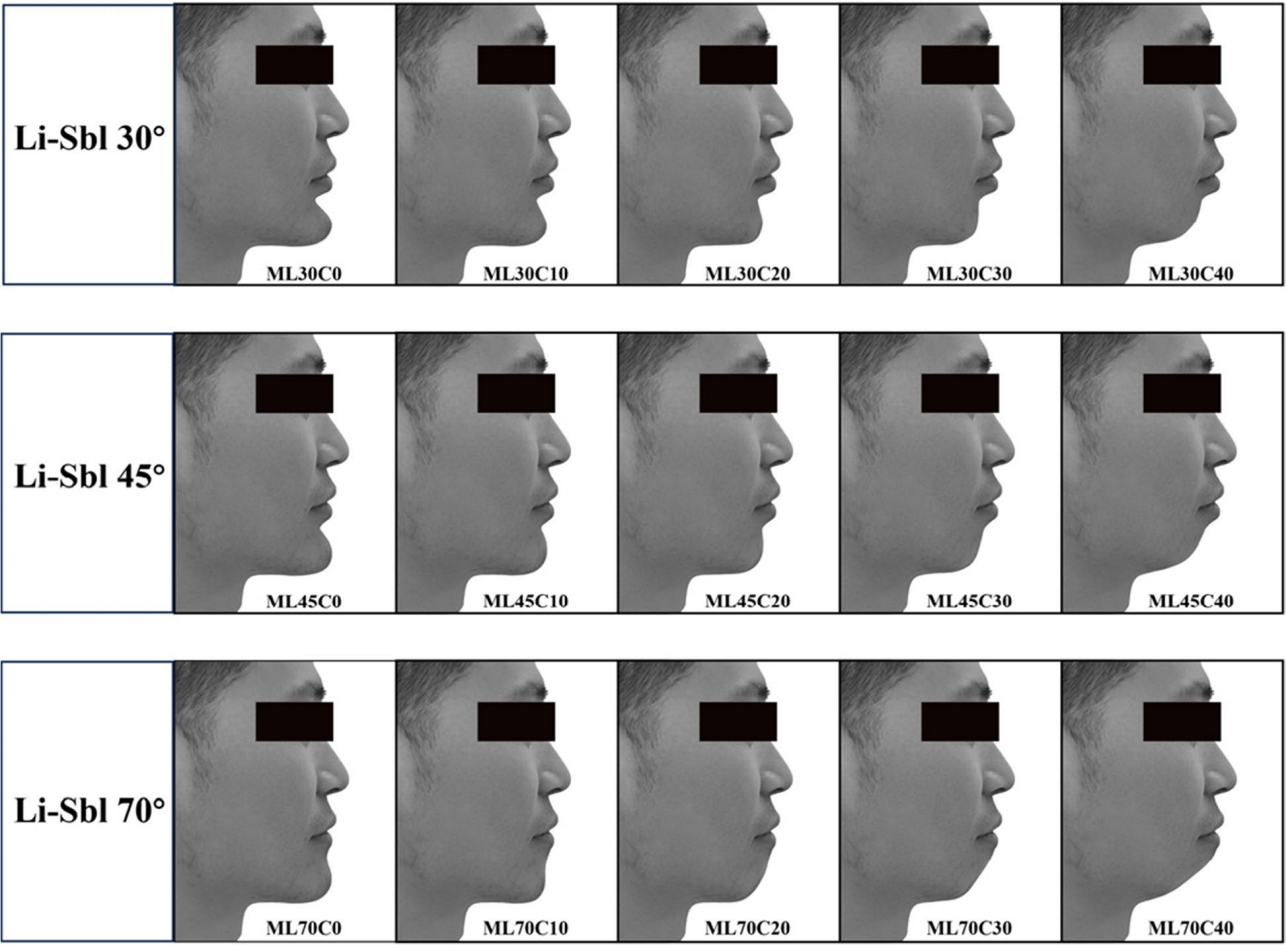
Male image configurations with varying Li-Sbl and Lia-Pog' angles. Fifteen standardized male images representing combinations of three Li-Sbl angles (30°, 45°, 70°) and five Lia-Pog' angles (0°, 10°, 20°, 30°, 40°). Image codes: ML30C0 to ML70C40 (M = male, L = Li-Sbl value, C = Lia-Pog' value)

All image manipulations were limited to the chin-lip region using Adobe Photoshop (v24.7). Target Sbl positions were determined by angular measurements from the fixed Li point (Li-Sbl angles: 30°, 45°, 70°), and target Pog' positions from the fixed Lia point (Lia-Pog' angles: 0° to 40°). Guide lines marking these target angles were established in Photoshop. The Liquify filter's Forward Warp Tool (brush size 50–150 pixels, density 50, pressure 30–50, rate 80) repositioned landmarks to calculated targets, followed by the Smooth Tool for natural blending. All manipulated images were reviewed by five experienced evaluators to ensure natural appearance without obvious digital manipulation artifacts. Images were converted to grayscale to eliminate color bias. Combining three Li-Sbl angles with five Lia-Pog' angles yielded 15 standardized images per gender. Female images were coded FL30C0 to FL70C40, male images ML30C0 to ML70C40 (Figs. 2 and 3).

### Recruitment and grouping of evaluators

#### Maxillofacial surgeons and orthodontists

Minimum 3 years of clinical experience in facial aesthetics.

#### Class II patients

Age ≥ 18 years, diagnosed with skeletal Class II malocclusion due to mandibular retrusion (ANB angle ≥ 5°), currently undergoing or committed to orthodontic treatment (having signed informed consent). All patients were informed about orthognathic surgery as a treatment option and expressed subjective concern about mandibular retrusion and facial aesthetics. Exclusion criteria included any history of orthognathic surgery, genioplasty, facial plastic surgery, or craniofacial trauma.

#### Laypeople

Age ≥ 18 years,no professional background in dentistry or related medical fields, no history of orthodontic or orthognathic treatment, and no apparent facial deformities or facial surgery history.

#### Sample size calculations

The required sample size for one-way ANOVA was determined using G*Power 3.1.9.7 software. Drawing from pilot data (*n* = 20/group) showing small to medium effect sizes (f≈0.18–0.35) and prior facial aesthetics studies [17, 18]. We conservatively estimated f = 0.25. Given the characteristically small effects in aesthetic perception research, with α = 0.05 and power = 0.85, the minimum sample size per group was calculated as 42. To maintain sufficient power for Bonferroni-corrected multiple comparisons, patient and layperson groups were expanded to 90 each, while professional groups were limited to 45 per specialty due to clinician availability constraints. The final sample comprised 45 maxillofacial surgeons, 45 orthodontists, 90 Class II patients, and 90 laypersons (*N* = 270), who were recruited offline from Shanghai Stomatological Hospital and affiliated clinics until target sample sizes were met.

### Aesthetic evaluation procedure

Before evaluation, participants completed a demographic questionnaire covering gender, age, and occupational background. Evaluators then received a brief introduction to the study objectives and scoring methods without disclosing specific structural parameters.

Evaluations occurred in a standardized environment (illuminance: 500 lx, color temperature: 5000 K). Initially, all 30 images were displayed simultaneously on one screen, allowing evaluators to gain a preliminary impression of aesthetic differences without revealing specific structural variations, minimizing cognitive bias.

During formal scoring, images appeared individually in random order on a 23-inch LCD monitor at approximately 70 cm viewing distance. Presentation order was randomized by computer (SPSS 26.0 random number generator). Female images were shown first, followed by male images, with randomized sequences within each gender category to minimize memory-related bias. Viewing time was unlimited, but evaluators could not revisit previously shown images.

Ratings utilized an 11-point numerical rating scale (NRS) ranging from 0 (extremely unattractive) to 10 (extremely attractive). Evaluators also recorded recommendations for surgical intervention (yes/no) for each facial configuration. To assess intra-rater reliability, 20% of evaluators were randomly selected to repeat the entire evaluation after four weeks under identical conditions.

### Statistical analysis

Statistical analyses were performed using SPSS version 26.0 (IBM, USA). Attractiveness scores were expressed as mean ± standard deviation (SD). Surgical recommendations were expressed as percentages. Between-group comparisons of attractiveness scores used the Kruskal–Wallis H test; surgical recommendation rates used chi-square or Fisher's exact tests (when expected frequencies < 5). Comparisons by gender and age (< 30 vs. ≥ 30 years) used the Mann–Whitney U test. Within-group aesthetic rankings used Wilcoxon signed-rank tests. Intra-rater consistency was assessed using ICC (two-way random, absolute agreement, 95% CI) for 20% of evaluators who completed repeat assessments. When significant differences emerged (*P* < 0.05), post-hoc pairwise comparisons were conducted using Bonferroni correction with adjusted significance levels: α = 0.008 for between-group comparisons (6 pairwise comparisons per configuration) and α = 0.0005 for within-group aesthetic rankings (105 pairwise comparisons). All tests were two-sided. In all tables, groups sharing the same superscript letters are not significantly different at the adjusted α level, and *P < 0.05 indicates statistical significance. Effect sizes were reported according to established conventions. For non-parametric tests: eta squared (η^2^) for Kruskal–Wallis test (0.01–0.06: small, 0.06–0.14: medium, > 0.14: large); r for Mann–Whitney U and Wilcoxon signed-rank tests, calculated as r = Z/√N (0.10–0.30: small, 0.30–0.50: medium, ≥ 0.50: large). For categorical comparisons: Cramér's V for chi-square tests and phi (φ) for 2 × 2 pairwise comparisons (0.10–0.30: small, 0.30–0.50: medium, ≥ 0.50: large).

## Results

### Study demographics

This study included 270 evaluators, comprising 103 males (38.1%) and 167 females (61.9%). Among them, 150 (55.6%) were under 30 years old, and 120 (44.4%) were aged 30 years or older. The evaluator groups included maxillofacial surgeons (*n* = 45), orthodontists (*n* = 45), Class II patients (*n* = 90), and laypeople (*n* = 90) (Table 1).

**Table 1:** Demographic characteristics of evaluators

Group	Number	Mean Age	95% CI (Age)	Male (%)	Age Range
Maxillofacial Surgeon	45	32.3	(30.7, 33.8)	64.4%	(23, 48)
Orthodontist	45	33.9	(31.9, 35.9)	28.8%	(23, 49)
Patient	90	25.7	(24.6, 26.9)	32.2%	(18, 40)
Laypeople	90	29.2	(28.0, 30.4)	35.6%	(22, 50)

#### Intra-rater reliability

Test–retest analysis showed moderate to good consistency across evaluator groups: maxillofacial surgeons (ICC = 0.82, 95% CI: 0.75–0.87), orthodontists (ICC = 0.78, 95% CI: 0.71–0.84), Class II patients (ICC = 0.71, 95% CI: 0.62–0.78), and laypeople (ICC = 0.74, 95% CI: 0.67–0.81).

### Aesthetic perception of female images

#### Li-Sbl 30

**Fig. 4:**
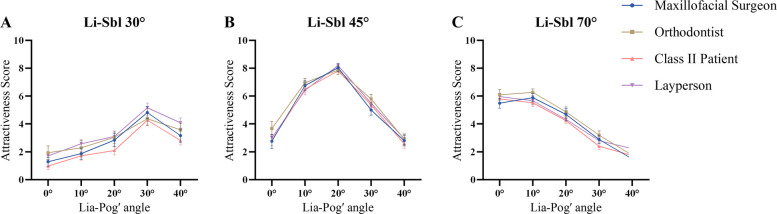
Aesthetic perception of female images across evaluator groups. Mean attractiveness scores with 95% CI for female image configurations at (**A**) Li-Sbl 30°, (**B**) Li-Sbl 45°, and (**C**) Li-Sbl 70°, evaluated by maxillofacial surgeons, orthodontists, Class II patients, and laypeople. Lia-Pog' angles ranged from 0° to 40° for each Li-Sbl category

All configurations received low attractiveness ratings (0.98–5.16), with significant inter-group differences (*P* ≤ 0.001; η^2^ = 0.052–0.093, medium to large effects). Patients scored lowest (0.98–4.27), showing clinically meaningful differences vs. orthodontists (0.58–1.01 points; *r* = 0.29–0.31) and laypeople (0.72–1.26 points; *r* = 0.27–0.37). FL30C20 and FL30C40 exceeded 1.0 point (1.01 and 1.26 points respectively, clinically significant), indicating heightened patient sensitivity to severe mandibular retrusion. FL30C30 scored highest (4.27–5.16) but remained below acceptable levels (Fig. 4A; Table 2; Supplementary Table 1).

**Table 2:** Comparison of attractiveness scores for female images among evaluator groups

Image	Maxillofacial Surgeon	Orthodontist	Class II patient	Laypeople	Effect Size (η^2^)	*P* value
FL30C0	1.29 ± 1.01^ab^	1.93 ± 1.62^a^	0.98 ± 1.19^b^	1.70 ± 1.37^a^	0.069	< 0.001*
FL30C10	1.87 ± 1.36^b^	2.29 ± 1.70^ab^	1.71 ± 1.55^b^	2.58 ± 1.48^a^	0.060	0.001*
FL30C20	2.84 ± 1.59^ab^	3.04 ± 1.49^a^	2.09 ± 1.51^b^	3.10 ± 1.48^a^	0.085	< 0.001*
FL30C30	4.82 ± 1.43^ab^	4.38 ± 1.63^ab^	4.27 ± 1.56^b^	5.16 ± 1.65^a^	0.052	0.001*
FL30C40	3.16 ± 1.55^b^	3.58 ± 1.53^ab^	2.82 ± 1.58^b^	4.08 ± 1.74^a^	0.093	< 0.001*
FL45C0	2.76 ± 1.68^a^	3.67 ± 1.75^a^	2.84 ± 1.85^a^	3.00 ± 1.03^a^	0.025	0.020*
FL45C10	6.76 ± 1.17	6.96 ± 1.02	6.50 ± 1.06	6.41 ± 1.56	0.011	0.081
FL45C20	8.04 ± 0.93	7.87 ± 0.99	7.81 ± 1.14	8.19 ± 0.82	0.008	0.059
FL45C30	5.00 ± 1.28^b^	5.80 ± 1.08^a^	5.38 ± 1.00^ab^	5.49 ± 1.21^ab^	0.036	0.010*
FL45C40	2.80 ± 1.27	2.91 ± 1.28	2.57 ± 1.48	2.94 ± 1.50	0.005	0.309
FL70C0	5.49 ± 1.16	6.09 ± 1.28	5.80 ± 1.28	5.97 ± 1.15	0.009	0.086
FL70C10	5.89 ± 0.98^ab^	6.27 ± 0.84^a^	5.52 ± 1.23^b^	5.67 ± 1.37^b^	0.057	0.005*
FL70C20	4.69 ± 1.43^ab^	4.87 ± 1.22^a^	4.24 ± 0.98^b^	4.33 ± 0.87^b^	0.036	0.005*
FL70C30	2.89 ± 1.01^ab^	3.20 ± 1.04^a^	2.40 ± 1.13^b^	2.80 ± 1.01^ab^	0.045	< 0.001*
FL70C40	1.49 ± 1.58^b^	1.76 ± 1.19^ab^	1.74 ± 1.31^ab^	2.23 ± 1.38^a^	0.036	0.013*

Values are mean ± SD. η^2^ = eta squared. Same letters indicate no significant difference (adjusted α = 0.008); "ab" is not significantly different from either "a" or "b" groups.

^*^P < 0.05

#### Li-Sbl 45

FL45C20 achieved highest scores (7.81–8.19) with negligible group effect (η^2^ = 0.008) and differences < 0.4 points, representing optimal aesthetic consensus. FL45C10 ranked second (6.41–6.96; η^2^ = 0.011). FL45C0 and FL45C30 showed small effects (η^2^ = 0.025–0.036), with orthodontists rating moderately higher than surgeons: FL45C0 (difference = 0.91 points, clinically relevant), FL45C30 (difference = 0.80 points, clinically relevant; *r* = 0.35). All differences remained below 1.0 point (Fig. 4B; Table 2; Supplementary Table 1).

#### Li-Sbl 70

Peak attractiveness at Lia-Pog' 0–10°. FL70C0 (5.49–6.09) showed negligible group differences (η^2^ = 0.009, < 0.6 points). FL70C10, FL70C20, FL70C30, and FL70C40 showed small effects (η^2^ = 0.036–0.057), with orthodontists rating 0.44–0.80 points higher than patients (*r* = 0.27–0.34). All differences remained below 1.0 point, indicating minimal impact of evaluator background when lower lip inclination is retroclined (Fig. 4C; Table 2; Supplementary Table 1).

**Fig. 5:**
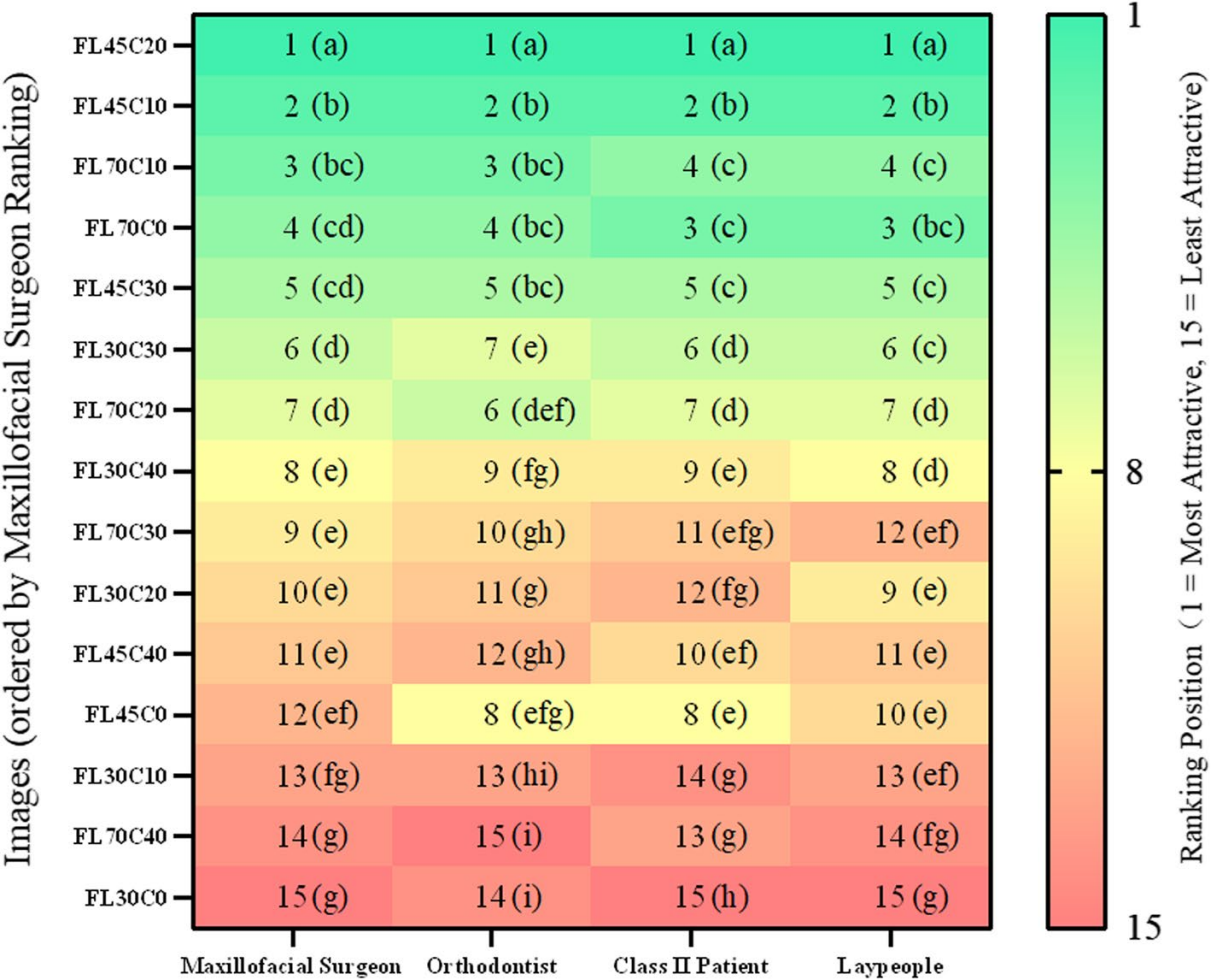
Aesthetic ranking of female images across evaluator groups. Rankings (1–15) ordered by maxillofacial surgeons' scores. Green to red gradient; same letters indicate no significant difference

FL45C20 ranked first across all groups, followed by FL45C10, FL70C0 and FL70C10. The least attractive were FL30C0, FL70C40, and FL30C10. All significant pairwise comparisons (α = 0.0005) showed at least medium effect sizes (r ≥ 0.3), with 96% showing large effects (r ≥ 0.5). Even between the top two configurations, differences were substantial: FL45C20 vs. FL45C10 (mean difference 0.91–1.78 points, *r* = 0.55–0.74, large effects), confirming clinically meaningful distinctions (Fig. 5; Supplementary Table 2).

### Aesthetic perception of male images

#### Li-Sbl 30

**Fig. 6:**
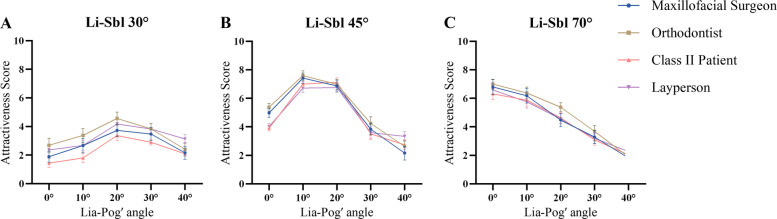
Aesthetic Perception of Male Images Across Evaluator Groups. Mean attractiveness scores with 95% CI for male image configurations at (**A**) Li-Sbl 30°, (**B**) Li-Sbl 45°, and (**C**) Li-Sbl 70°, evaluated by maxillofacial surgeons, orthodontists, Class II patients, and laypeople. Lia-Pog' angles ranged from 0° to 40° within each Li-Sbl category

All configurations received low ratings (1.44–4.56), with significant inter-group differences (*P* ≤ 0.003; η^2^ = 0.044–0.098, medium to large effects). Patients scored lowest (1.44–3.37), showing clinically meaningful differences vs. orthodontists (0.94–1.58 points; *r* = 0.32–0.43) and laypeople (0.59–0.93 points; *r* = 0.25–0.31). ML30C10 (difference = 1.58 points, highly clinically significant) and ML30C0 (difference = 1.24 points, clinically significant) exceeded 1.0 point, confirming heightened patient sensitivity. ML30C20 scored highest (3.37–4.56) but remained below acceptable levels (Fig. 6A; Table 3; Supplementary Table 3).

**Table 3:** Comparison of attractiveness scores for male images among evaluator groups

Image	Maxillofacial Surgeon	Orthodontist	Class II patient	Laypeople	Effect Size (η^2^)	P value
ML30C0	1.89 ± 1.27^ab^	2.69 ± 1.64^a^	1.44 ± 1.44^b^	2.36 ± 1.93^a^	0.065	< 0.001*
ML30C10	2.67 ± 1.68^a^	3.38 ± 1.61^a^	1.80 ± 1.59^b^	2.68 ± 1.79^a^	0.098	< 0.001*
ML30C20	3.73 ± 1.39^ab^	4.56 ± 1.52^a^	3.37 ± 1.74^b^	4.17 ± 2.39^ab^	0.044	0.003*
ML30C30	3.47 ± 1.55^ab^	3.84 ± 1.26^a^	2.90 ± 0.94^b^	3.83 ± 1.84^a^	0.079	< 0.001*
ML30C40	2.16 ± 1.51^b^	2.40 ± 1.54^ab^	2.09 ± 1.86^b^	3.13 ± 1.48^a^	0.075	< 0.001*
ML45C0	4.98 ± 1.03^a^	5.38 ± 0.89^a^	3.91 ± 1.00^b^	4.02 ± 0.98	0.086	< 0.001*
ML45C10	7.42 ± 1.16^a^	7.60 ± 1.12^a^	7.00 ± 0.89^b^	6.72 ± 1.46^b^	0.053	< 0.001*
ML45C20	6.87 ± 1.38	6.98 ± 1.22	7.13 ± 1.52	6.76 ± 1.10	0.019	0.279
ML45C30	3.84 ± 1.38	4.24 ± 1.51	3.51 ± 1.94	3.60 ± 1.92	0.007	0.128
ML45C40	2.16 ± 1.62^b^	2.62 ± 1.43^ab^	2.74 ± 1.63^ab^	3.33 ± 1.59^a^	0.051	< 0.001*
ML70C0	6.80 ± 1.70	7.00 ± 1.11	6.31 ± 1.90	6.59 ± 1.71	0.002	0.126
ML70C10	6.18 ± 1.70	6.38 ± 1.35	5.87 ± 2.17	5.76 ± 2.11	0.000	0.289
ML70C20	4.47 ± 1.56	5.38 ± 1.07	4.62 ± 1.88	4.56 ± 1.69	0.026	0.263
ML70C30	3.29 ± 1.55	3.69 ± 1.38	3.09 ± 1.79	3.19 ± 1.70	0.004	0.248
ML70C40	1.82 ± 1.60	1.89 ± 1.19	2.03 ± 1.56	2.27 ± 1.73	0.000	0.374

Values are mean ± SD. η^2^ = eta squared. Same letters indicate no significant difference (adjusted α = 0.008); "ab" is not significantly different from either "a" or "b" groups.

^*^P < 0.05

#### Li-Sbl 45

ML45C10 (6.72–7.60) and ML45C20 (6.76–7.13) achieved optimal scores with negligible to small effects (η^2^ = 0.019–0.053) and clinically insignificant differences (< 0.7 points), representing strong aesthetic consensus. ML45C0 showed medium effect (η^2^ = 0.085), with professionals rating 1.07–1.47 points higher than patients/laypeople (approaching or exceeding clinical significance; *r* = 0.32–0.35). Most other differences remained below 1.0 point (clinically relevant), substantially less than Li-Sbl 30° (Fig. 6B; Table 3; Supplementary Table 3).

#### Li-Sbl 70

Peak attractiveness at Lia-Pog' 0–10°. ML70C0 (6.31–7.00) and ML70C10 (5.76–6.38) received optimal scores with negligible group differences (η^2^ ≤ 0.002) and clinically insignificant variation (< 0.5 points), indicating strong aesthetic consensus. ML70C20 showed small effect (η^2^ = 0.026) with differences < 0.9 points (clinically relevant), while ML70C30 and ML70C40 showed negligible effects (η^2^ ≤ 0.004) with minimal variation (< 0.6 points). No comparisons exceeded 1.0 point (Fig. 6C; Table 3; Supplementary Table 3).

**Fig. 7:**
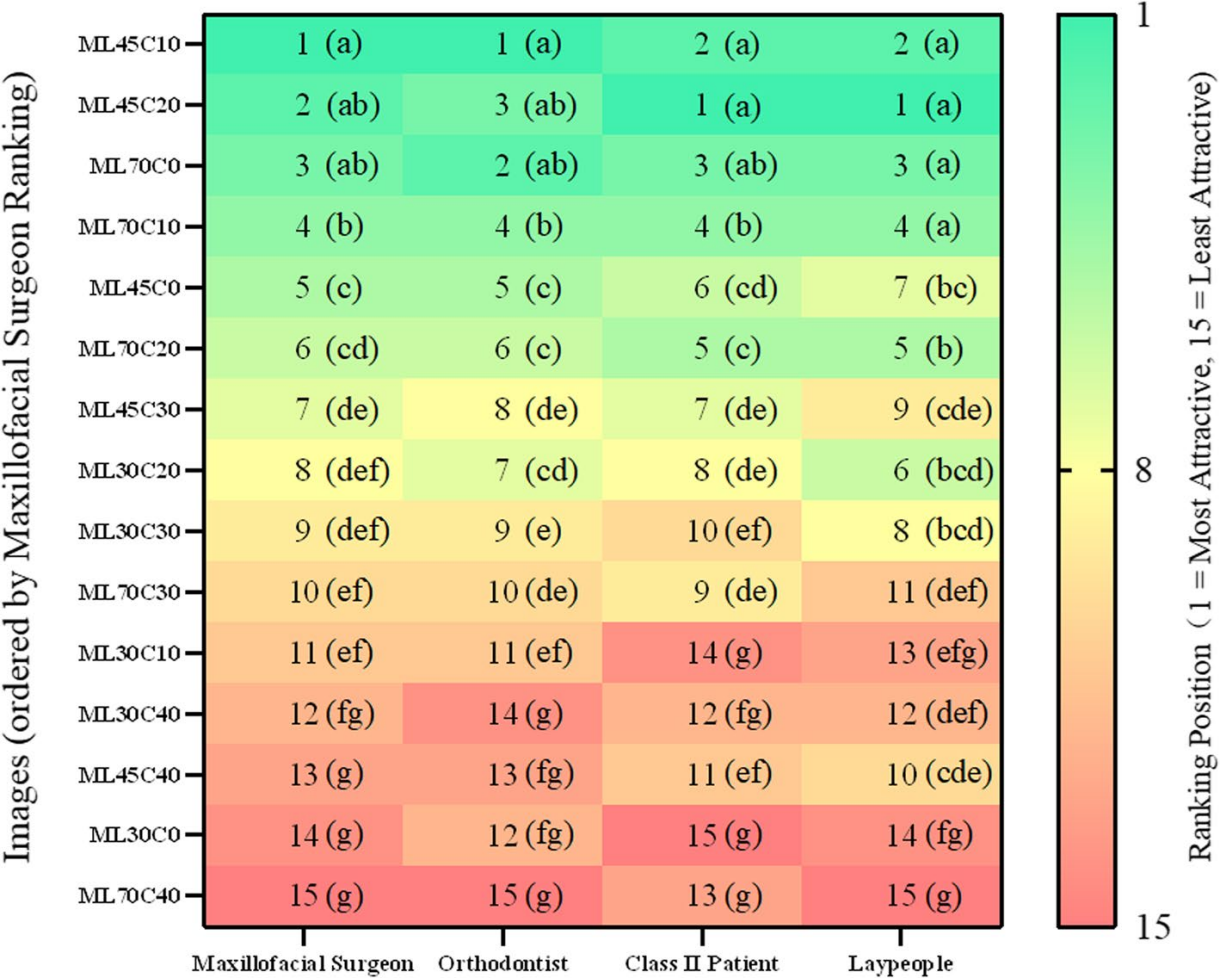
Aesthetic ranking of male images across evaluator groups. Rankings (1–15) ordered by maxillofacial surgeons' scores. Green to red gradient; same letters indicate no significant difference

ML45C10, ML45C20, and ML70C0 ranked highest among surgeons, orthodontists, and Class II patients. Laypeople also rated ML70C10 equally high. The lowest-rated were ML70C40 and ML30C0. All significant pairwise comparisons (α = 0.0005) showed at least medium effect sizes (r ≥ 0.3), with 93% showing large effects (r ≥ 0.5), demonstrating clinically relevant differences (Fig. 7; Supplementary Table 4).

### Surgical recommendations for female images

#### Li-Sbl 30

**Fig. 8:**
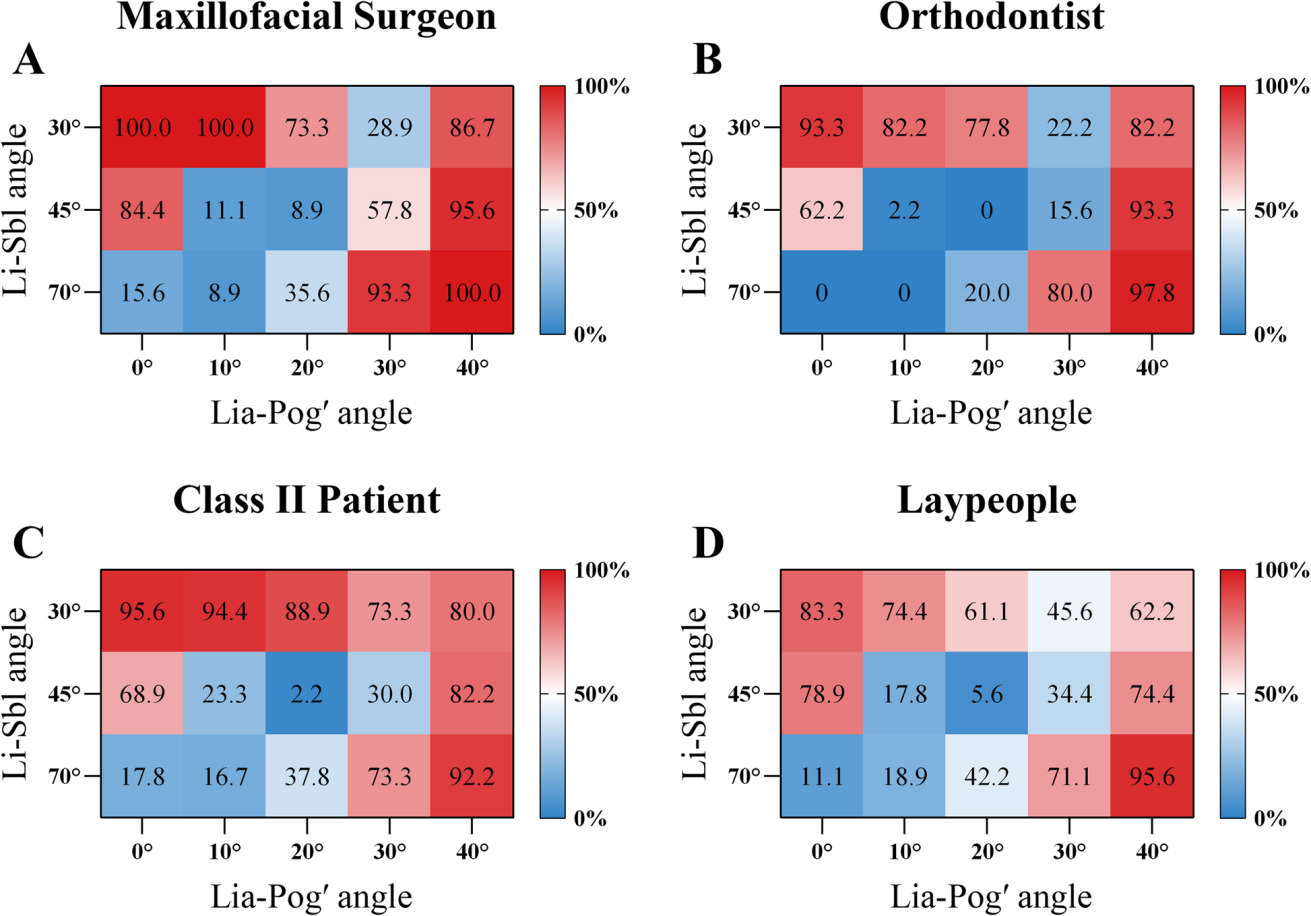
Surgical treatment preferences for female images. Surgical recommendation rates (%) for female image configurations across evaluator groups at (**A**) maxillofacial surgeons, (**B**) orthodontists, (**C**) Class II patients, and **(D)** laypeople. Heat maps show recommendation percentages for each combination of Li-Sbl angle (30°, 45°, 70°) and Lia-Pog' angle (0°, 10°, 20°, 30°, 40°), with red indicating higher and blue indicating lower recommendation rates

FL30C0, FL30C10, FL30C20, and FL30C40 received majority surgical recommendations (> 50% across all groups; Cramér's V = 0.225–0.298). FL30C30 demonstrated substantial inter-group divergence (Cramér's V = 0.394; *P* < 0.001), with only Class II patients recommending surgery at majority rates (73.3%), while clinicians (< 29%) and laypeople (45.6%) predominantly opposed surgical intervention (*P* < 0.001 vs. patients, φ = 0.409–0.470) (Fig. 8; Table 4).

**Table 4:** Surgical recommendation rates for female images among evaluator groups

Image	Maxillofacial Surgeon	Orthodontist	Class II patient	Laypeople	Effect Size (Cramér's V)	*P* value
FL30C0	100.0%	93.3%^ab^	95.6%^ab^	83.3%^b^	0.232	0.002*
FL30C10	100.0%^a^	82.2%^b^	94.4%^a^	74.4%^b^	0.298	< 0.001*
FL30C20	73.3%^ab^	77.8%^ab^	88.9%^a^	61.1%^b^	0.264	< 0.001*
FL30C30	28.9%^b^	22.2%^b^	73.3%^a^	45.6%^b^	0.394	< 0.001*
FL30C40	86.7%^a^	82.2%^ab^	80.0%^ab^	62.2%^b^	0.225	0.003*
FL45C0	84.4%	62.2%	68.9%	78.9%	0.173	0.055
FL45C10	11.1%^ab^	2.2%^b^	23.3%^a^	17.8%^ab^	0.202	0.012*
FL45C20	8.9%	0.0%	2.2%	5.6%	0.148	0.118
FL45C30	57.8%^a^	15.6%^b^	30.0%^b^	34.4%^b^	0.264	< 0.001*
FL45C40	95.6%^a^	93.3%^ab^	82.2%^ab^	74.4%^b^	0.224	0.004*
FL70C0	15.6%^a^	0.0%^b^	17.8%^a^	11.1%^ab^	0.187	0.024*
FL70C10	8.9%^ab^	0.0%^b^	16.7%^a^	18.9%^a^	0.201	0.012*
FL70C20	35.6%	20.0%	37.8%	42.2%	0.157	0.084
FL70C30	93.3%^a^	80.0%^ab^	73.3%^b^	71.1%^b^	0.187	0.024*
FL70C40	100.0%	97.8%	92.2%	95.6%	0.136	0.174

Values are percentages. Same letters indicate no significant difference (adjusted α = 0.008); "ab" is not significantly different from either "a" or "b" groups.

^*^P < 0.05

#### Li-Sbl 45

FL45C10 and FL45C20 received minimal surgical recommendations (0–23.3%; Cramér's V = 0.148–0.202). FL45C0 and FL45C40 received majority recommendations (Cramér's V = 0.173 and 0.224). FL45C30 demonstrated substantial inter-group disagreement (Cramér's V = 0.264; *P* < 0.001), with only surgeons recommending surgery at majority rates (57.8%), while other groups (< 35%) predominantly opposed intervention (*P* < 0.001 vs. surgeons, φ = 0.207–0.415) (Fig. 8; Table 4).

#### Li-Sbl 70

FL70C0 and FL70C10 received minimal surgical recommendations (< 20%; Cramér's V = 0.187–0.201), while FL70C30 and FL70C40 showed high consensus for surgery (Cramér's V = 0.136–0.187). FL70C20 showed moderate recommendations (20.0–42.2%) with no significant inter-group differences (*P* = 0.084; Cramér's V = 0.157) (Fig. 8; Table 4).

### Surgical recommendations for male images

#### Li-Sbl 30

**Fig. 9:**
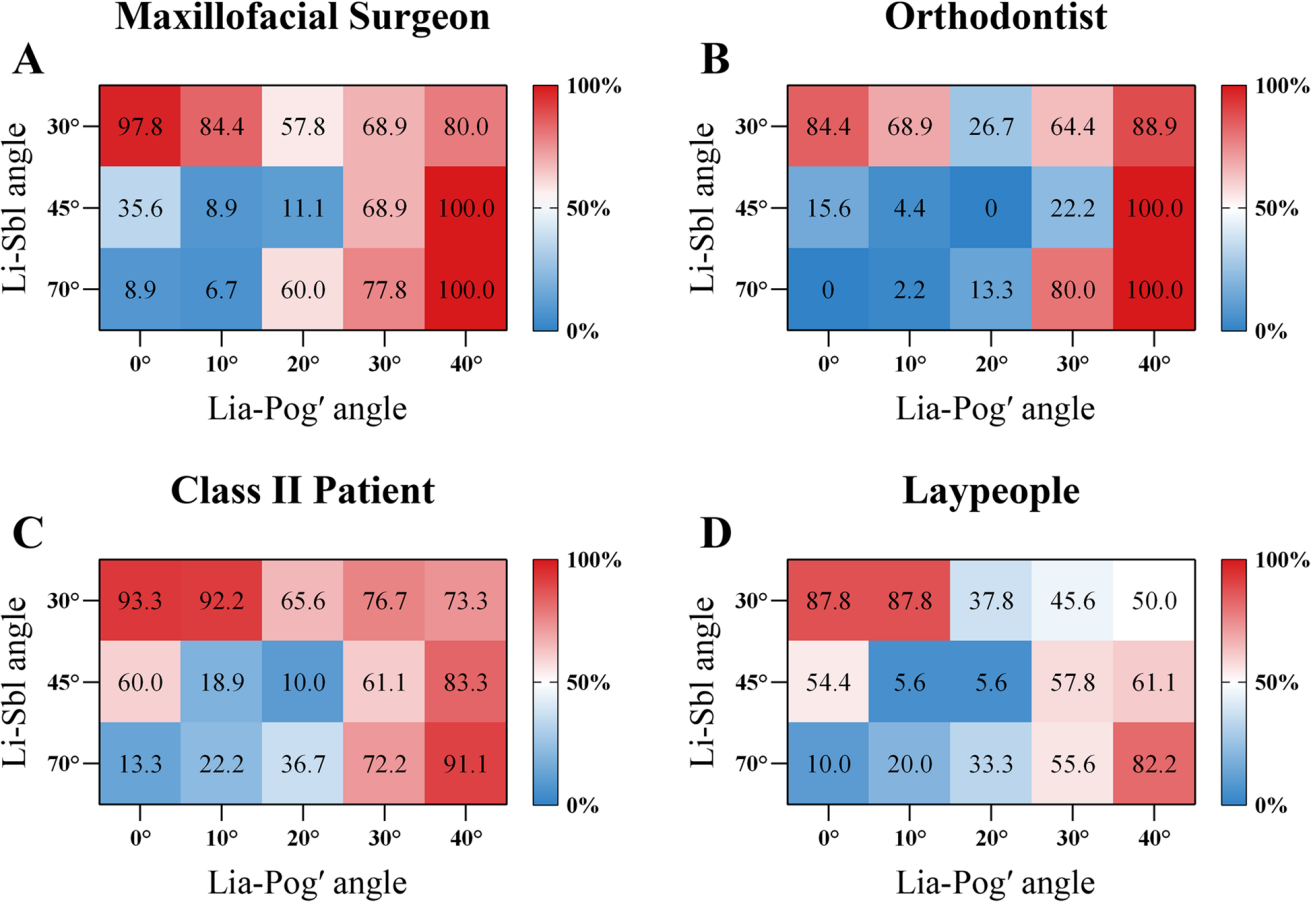
Surgical treatment preferences for male images. Surgical recommendation rates (%) for male image configurations across evaluator groups at (**A**) maxillofacial surgeons, (**B**) orthodontists, (**C**) Class II patients, and (**D**) laypeople. Heat maps show recommendation percentages for each combination of Li-Sbl angle (30°, 45°, 70°) and Lia-Pog' angle (0°, 10°, 20°, 30°, 40°), with red indicating higher and blue indicating lower recommendation rates

ML30C0 showed high consensus for surgery (84.4–97.8%; Cramér's V = 0.154). ML30C10 and ML30C40 received majority recommendations (Cramér's V = 0.226 and 0.316). ML30C20 and ML30C30 demonstrated substantial inter-group divergence (Cramér's V = 0.303 and 0.270; both *P* < 0.001), with recommendation rates crossing the 50% threshold. For ML30C20: surgeons and patients (57.8–65.6%) versus orthodontists and laypeople (< 38%), with orthodontists showing the lowest recommendation (26.7%; *P* < 0.001 vs. patients, φ = 0.351). For ML30C30: clinicians and patients (64.4–76.7%) versus laypeople (45.6%) (Fig. 9; Table 5).

**Table 5:** Surgical recommendation rates for male images among evaluator groups

Image	Maxillofacial Surgeon	Orthodontist	Class II patient	Laypeople	Effect Size (Cramér's V)	*P* value
ML30C0	97.8%	84.4%	93.3%	87.8%	0.154	0.09
ML30C10	84.4%^ab^	68.9%^b^	92.2%^a^	87.8%^ab^	0.226	0.003*
ML30C20	57.8%^a^	26.7%^b^	65.6%^a^	37.8%^b^	0.303	< 0.001*
ML30C30	68.9%^b^	64.4%^ab^	76.7%^a^	45.6%^b^	0.270	< 0.001*
ML30C40	80.0%^a^	88.9%^a^	73.3%^a^	50.0%^b^	0.316	< 0.001*
ML45C0	35.6%^ab^	15.6%^b^	60.0%^a^	54.4%^a^	0.324	< 0.001*
ML45C10	8.9%^ab^	4.4%^ab^	18.9%^a^	5.6%^b^	0.203	0.011*
ML45C20	11.1%	0.0%	10.0%	5.6%	0.150	0.108
ML45C30	68.9%^a^	22.2%^b^	61.1%^a^	57.8%^a^	0.302	< 0.001*
ML45C40	100.0%^a^	86.7%^ab^	83.3%^b^	61.1%^c^	0.345	< 0.001*
ML70C0	8.9%	0.0%	13.3%	10.0%	0.154	0.092
ML70C10	6.7%^ab^	2.2%^b^	22.2%^b^	20.0%^b^	0.221	0.004*
ML70C20	60.0%^a^	13.3%^b^	36.7%^a^	33.3%^a^	0.283	< 0.001*
ML70C30	77.8%^ab^	80.0%^a^	72.2%^ab^	55.6%^b^	0.212	0.006*
ML70C40	100.0%^a^	100.0%^a^	91.1%^ab^	82.2%^b^	0.255	< 0.001*

Values are percentages. Same letters indicate no significant difference (adjusted α = 0.008); "ab" is not significantly different from either "a" or "b" groups.

^*^P < 0.05

#### Li-Sbl 45

ML45C10 and ML45C20 received minimal surgical recommendations (0–18.9%; Cramér's V = 0.150–0.203). ML45C40 showed majority recommendations (Cramér's V = 0.345). ML45C0 and ML45C30 demonstrated substantial inter-group divergence (Cramér's V = 0.324 and 0.302; both *P* < 0.001), with recommendation rates crossing the 50% threshold. For ML45C0: patients (60.0%) and laypeople (54.4%) versus clinicians (< 36%), with orthodontists showing the lowest recommendation (15.6%; *P* < 0.001 vs. patients, φ = 0.405). For ML45C30: clinicians and patients (61.1–68.9%) versus orthodontists (22.2%; *P* < 0.001, φ = 0.321–0.446) (Fig. 9; Table 5).

#### Li-Sbl 70

ML70C0 and ML70C10 received minimal surgical recommendations (< 23%; Cramér's V = 0.154–0.221). ML70C20 demonstrated substantial inter-group divergence (Cramér's V = 0.283; *P* < 0.001), with only surgeons recommending surgery at majority rates (60.0%), while other groups (< 37%) predominantly opposed intervention (*P* < 0.001 vs. surgeons, φ = 0.239–0.461). ML70C30 and ML70C40 showed majority recommendations (Cramér's V = 0.212 and 0.255) (Fig. 9; Table 5).

### Evaluator age and gender effects

Evaluator age showed modest effects while gender had minimal influence. For attractiveness ratings, older evaluators (≥ 30 years) assigned slightly higher scores in 5 female and 5 male images, with mean differences of 0.39–0.69 points (small effects), indicating minimal clinical impact on treatment planning decisions. For surgical recommendations, age showed small effects in 2 female images (FL30C10, FL30C20) but no significant effects in male images. Gender effects were negligible across all configurations (Supplementary Tables 5–12).

## Discussion

### Principal findings and clinical significance

This study systematically investigated the interactive effects of Li-Sbl and Lia-Pog' angles on facial aesthetics, revealing significant gender-specific patterns and compensation mechanisms. Females required a narrow optimal range: Li-Sbl 45° with Lia-Pog′ 20° (scores 7.81–8.19). Males demonstrated broader tolerance, with three optimal configurations: Li-Sbl 45° with Lia-Pog′ 10°−20°, or Li-Sbl 70° with Lia-Pog′ 0° (scores 6.31–7.60). Changes in Li-Sbl angle significantly altered the distribution of optimal chin positions. Critically, when Li-Sbl ≤ 30°, aesthetic scores remained low despite optimized chin positioning, indicating that isolated genioplasty cannot compensate for severe lower lip retrusion.

Evaluator professional background substantially influenced aesthetic perception and treatment recommendations. Class II patients demonstrated heightened sensitivity to severe lower lip retrusion (Li-Sbl 30°), rating these configurations significantly lower than orthodontists and laypeople. For controversial configurations, maxillofacial surgeons and Class II patients favored surgical intervention, while orthodontists adopted conservative approaches.

Based on these findings, we developed a clinical decision support system that addresses two sequential decision points through quantitative thresholds. The first decision point determines whether surgical intervention is necessary, providing systematic criteria to identify borderline cases requiring multidisciplinary consultation. The second decision point, when intervention is indicated, determines which surgical approach is appropriate. The Li-Sbl angle serves as the critical threshold: when Li-Sbl ≤ 30°, isolated genioplasty shows insufficient efficacy, indicating comprehensive orthognathic surgery is required; when Li-Sbl > 30°, isolated genioplasty becomes technically feasible. The Li-Sbl × Lia-Pog' model evaluates two surgically modifiable components: lower lip inclination and chin prominence. Their interactive configurations guide both decision points. Gender-specific visualization charts (Figs. 2, 3) further enhance clinical utility. This framework transforms subjective clinical judgment into evidence-based decision criteria, facilitating informed consent, enhancing patient understanding, and optimizing treatment planning.

### Surgical applications and treatment algorithms

Understanding the surgical modifiability of Li-Sbl and Lia-Pog' angles is fundamental for translating research findings into clinical practice. The Li-Sbl angle reflects the overall sagittal relationship between the mandible and lower lip. Its modification depends on orthodontic-orthognathic treatment to alter lower incisor inclination and mandibular position. Isolated genioplasty has minimal impact on Li-Sbl [19]. In contrast, the Lia-Pog' angle represents chin prominence relative to the lower lip. It can be directly adjusted through genioplasty. This difference in surgical modifiability provides a theoretical basis for individualized treatment planning.

In clinical practice, measuring Li-Sbl and Lia-Pog' angles is straightforward. Clinicians can use a simple protractor with the patient in natural head position, without requiring complex cephalometric equipment. For standardized measurement, the patient should be relaxed in rest position with lips naturally closed.

For patients with an unclear mentolabial sulcus, a practical technique is to gently push the chin soft tissue forward with a finger to simulate genioplasty. This deepens the mentolabial sulcus, allowing accurate identification of Sbl for Li-Sbl measurement while enabling the patient to preview potential aesthetic changes [20].

Accurate measurement requires understanding the anatomical basis and functional influences. Lower lip morphology is not isolated but comprehensively influenced by multiple factors, including lower incisor inclination, anterior tooth overjet, lower facial vertical height, and lip function. Excessively proclined lower incisors or severe deep bite cause excessive lower lip eversion. Insufficient lower facial height compresses soft tissue, decreasing Li-Sbl and deepening the mentolabial sulcus. Lip incompetence leads to compensatory chin muscle tension and soft tissue prominence [3, 21, 22]. These changes all affect the measurement accuracy of Li-Sbl and Lia-Pog' angles.

Therefore, when measuring Li-Sbl and Lia-Pog' angles, clinicians should simultaneously assess the following through lateral cephalometric measurement and clinical examination: (1) lower facial vertical height; (2) lower incisor inclination and tooth overjet; (3) lip closure function. By identifying and analyzing these factors, clinicians can accurately determine the underlying causes of soft tissue abnormalities and develop targeted treatment plans. Emphasizing the functional priority principle is particularly important. Treatment plans should not be based solely on aesthetic parameters but should prioritize correction of functional disorders, including masticatory dysfunction, speech disorders, obstructive sleep apnea, and temporomandibular joint disorders [23].

#### Clinical protocol for Li-Sbl × Lia-Pog' assessment and treatment planning

**Fig. 10:**
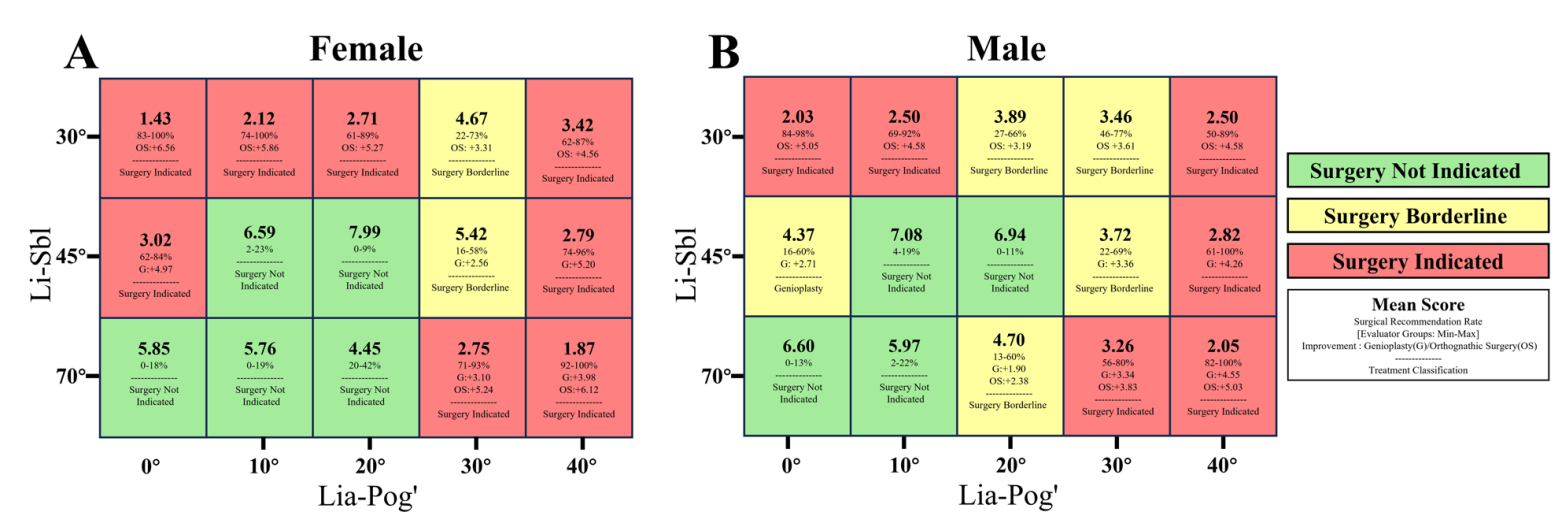
Clinical decision matrix for Li-Sbl × Lia-Pog' assessment. Heat maps for (**A**) female and (**B**) male patients (*n* = 270 evaluators). Each cell: mean score, surgical recommendation rate (%), treatment classification. Color coding: green = surgery not indicated; yellow = borderline cases; red = surgery indicated. Optimal configurations: females Li-Sbl 45°/Lia-Pog' 20° (7.99); males Li-Sbl 45°/Lia-Pog' 10°−20° (6.94–7.08) or Li-Sbl 70°/Lia-Pog' 0° (6.60)

Based on data from 270 evaluators, we developed a clinical decision support system for Li-Sbl × Lia-Pog' combinations in the Chinese population (Fig. 10). For each configuration, this system provides: (1) mean aesthetic score; (2) surgical recommendation rate (%); (3) improvement potential; (4) treatment classification. The decision matrix provides systematic guidance through two sequential decision points. These address whether intervention is necessary and what surgical approach is feasible.

The first decision point addresses whether any surgical intervention is warranted. Borderline cases are defined as configurations where surgical recommendation rates cross the 50% threshold between evaluator groups, with at least one pairwise comparison showing medium effect size (φ ≥ 0.30, *P* < 0.008). Seven configurations met these criteria: FL30C30, FL45C30, ML30C20, ML30C30, ML45C0, ML45C30, and ML70C20 (yellow cells in Fig. 10). For these borderline configurations, intervention necessity itself remains contested. Maxillofacial surgeons, orthodontists, Class II patients, and laypeople show significant variation in surgical recommendations. These cases require multidisciplinary consultation. The consultation should integrate aesthetic predictions, functional assessments, and patient expectations. This determines whether surgical intervention is warranted.

The second decision point determines treatment feasibility for patients where intervention is indicated. It answers whether isolated genioplasty is sufficient or comprehensive orthognathic surgery is required. The Li-Sbl angle serves as the critical threshold. When Li-Sbl ≤ 30°, isolated genioplasty shows limited improvement. Even with optimal chin positioning, mean aesthetic scores remain low (females: 4.67, males: 3.89). These scores fall within the borderline zone. They cannot reach the "Surgery Not Indicated" (green) threshold. These patients need comprehensive orthodontic-orthognathic treatment. This represents the most fundamental clinical decision applicable to all patients requiring intervention. In contrast, when Li-Sbl reaches 45° or higher, isolated genioplasty can achieve optimal outcomes. For females, aesthetic scores reach 7.99 at Li-Sbl 45°/Lia-Pog' 20°. For males, scores reach 6.94–7.08 at Li-Sbl 45°/Lia-Pog' 10°−20°. For select cases with Li-Sbl 70°, both treatment modalities may produce acceptable outcomes. In these cases, clinicians can further compare the magnitude of aesthetic improvement. This guides treatment selection based on patient-centered decision-making.

To implement this system in clinical practice, clinicians should follow a six-step protocol:

1. Comprehensive assessment: Evaluate skeletal structures, dental relationships, soft tissue characteristics, and functional concerns through lateral cephalometry and clinical examination. These factors influence Li-Sbl and Lia-Pog' measurements.
2. Angular measurement: Measure Li-Sbl and Lia-Pog' angles accurately with the patient in natural head position, rest position, and lips naturally closed.
3. Matrix consultation: Locate patient's configuration in the corresponding gender table (Fig. 10) to obtain aesthetic score, surgical recommendation rate, improvement potential, and color-coded classification. For patients with Li-Sbl between tested angles, use the following guidelines: values closer to 30° favor orthognathic surgery; Li-Sbl 40°−60° should reference 45° data; Li-Sbl > 65° should reference 70° data.
4. Decision point navigation: First, if the configuration falls in a yellow cell (borderline case), arrange multidisciplinary consultation considering aesthetic scores, functional status, and patient concerns. Second, determine treatment modality feasibility using the Li-Sbl threshold: if Li-Sbl ≤ 30°, comprehensive orthodontic-orthognathic surgery is indicated; if Li-Sbl > 30°, isolated genioplasty is technically feasible. For cases where both modalities are viable, compare predicted aesthetic improvements based on patient expectations and functional needs.
5. Surgical simulation: Perform simulation using lateral radiographs or 3D imaging to determine movement magnitude and verify alignment with matrix predictions.
6. Feasibility verification: Ensure horizontal genioplasty advancement does not exceed anatomical limits (typically ≤ 10–12 mm) [24]. For severe chin retrusion (Lia-Pog' ≥ 40°), consider combined orthognathic surgery or double-sliding genioplasty when required movement exceeds this range [25].

A standardized clinical documentation template is provided in Supplementary Material 1.

#### Clinical case examples

**Fig. 11:**
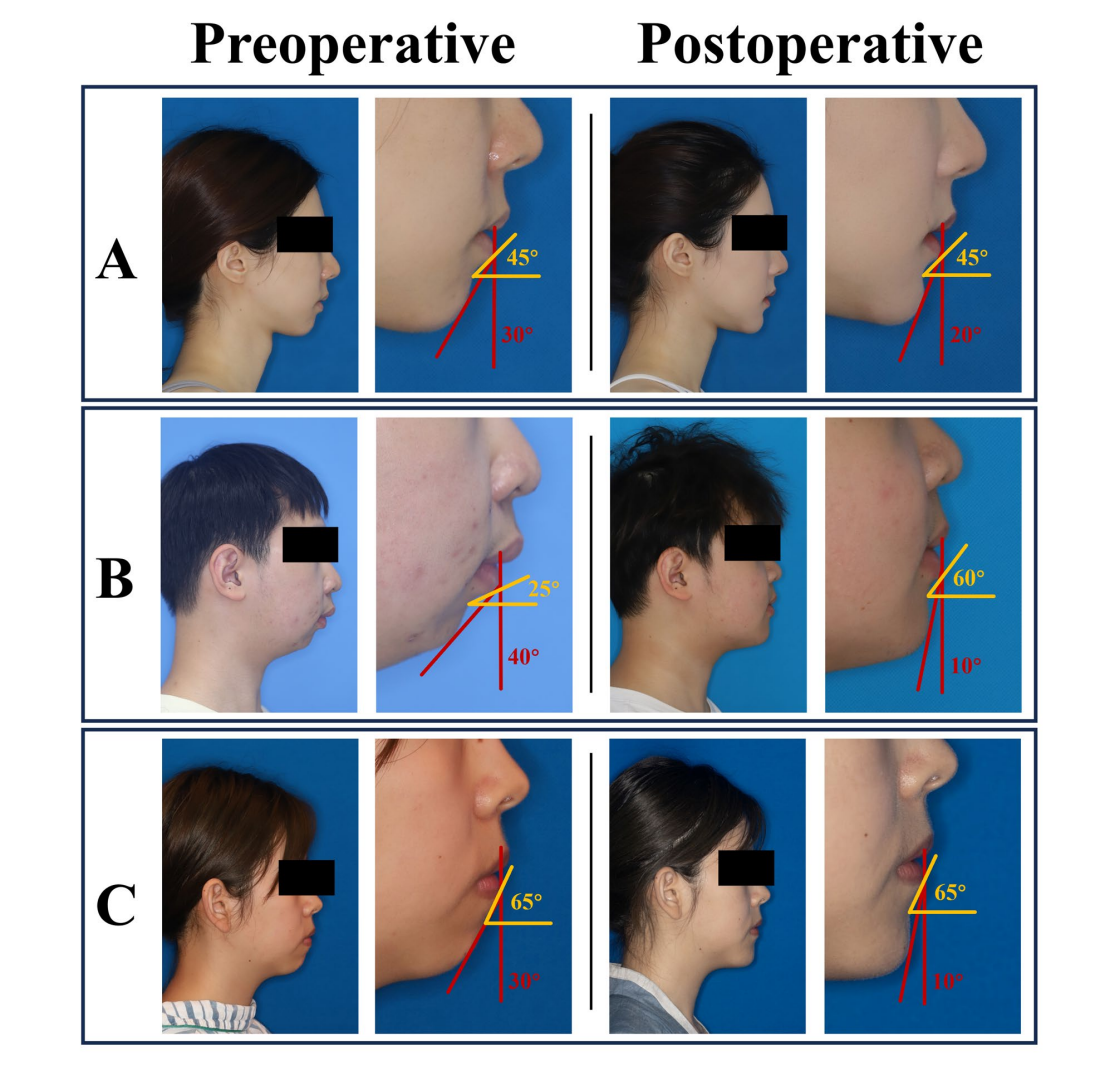
Clinical application of the decision framework. **A** Female, Li-Sbl 45°, Lia-Pog' 30° → 20° (Surgery Borderline): isolated genioplasty after consultation. **B** Male, Li-Sbl 25° → 65°, Lia-Pog' 40° → 10° (Surgery Indicated): comprehensive orthognathic surgery (Li-Sbl ≤ 30°). **C** Female, Li-Sbl 65°, Lia-Pog' 30° → 10° (Surgery Indicated): isolated genioplasty chosen (Li-Sbl ≥ 30°, either approach viable). Yellow arrows = Li-Sbl; red arrows = Lia-Pog'

Three cases illustrate the clinical decision framework (Fig. 11).

##### **Case 1**

Female, Li-Sbl 45°, Lia-Pog' 30° (Surgery Borderline). Multidisciplinary consultation determined intervention was warranted. After discussion with the patient, surgical treatment was decided. Isolated genioplasty improved Lia-Pog' to 20° (Fig. 11A).

##### **Case 2**

Male, Li-Sbl 25°, Lia-Pog' 40° (Surgery Indicated). Li-Sbl ≤ 30° indicated comprehensive orthodontic-orthognathic surgery required. Treatment improved Li-Sbl to 65° and Lia-Pog' to 10° (Fig. 11B).

##### **Case 3**

Female, Li-Sbl 65°, Lia-Pog' 30° (Surgery Indicated). With Li-Sbl ≥ 30°, both orthognathic surgery and genioplasty could achieve the "Surgery Not Indicated" zone. After assessing patient expectations and weighing treatment complexity, patient chose isolated genioplasty, improving Lia-Pog' to 10° (Fig. 11C).

#### Surgical considerations and complication management

The Li-Sbl angle also serves an important role in complication prevention. Beyond guiding treatment decisions, the preoperative Li-Sbl angle serves as a key predictor of excessive mentolabial sulcus depth—a common aesthetic complication after genioplasty [26]. The Li-Sbl angle reflects the depth of the upper mentolabial sulcus. Smaller Li-Sbl angles indicate a proclined, everted lower lip position, which creates a deeper upper sulcus. Chin advancement then deepens the lower segment, resulting in overall excessive sulcus depth. For patients with Li-Sbl ≤ 30°, the upper mentolabial sulcus is already markedly deep. Isolated genioplasty fails to improve aesthetic scores and may even worsen them. For patients with Li-Sbl 45°, advancement magnitude must be carefully controlled. For females, optimal Lia-Pog' is 20° (mean score 7.99). Advancement to 10° decreases the score to 6.59. Further advancement to 0° decreases the score to 3.02. For males, Lia-Pog' 0° (mean score 4.37) is significantly lower than 10°−20° (mean scores 6.94–7.08). For patients with Li-Sbl ≥ 70°, advancement magnitude can be increased appropriately, but anatomical limits and individual differences must be noted.

Soft tissue variability and functional outcomes also affect postoperative results. This study used idealized profile images to establish aesthetic benchmarks. This allows standardized evaluation. However, it cannot fully capture individual soft tissue variability. Soft tissue characteristics vary considerably among patients. These differences may cause actual outcomes to deviate from predicted scores [27]. Therefore, the Li-Sbl × Lia-Pog' predictions represent theoretical targets. Surgeons must integrate these predictions with individualized soft tissue assessment.

More importantly, functional outcomes should take priority over aesthetic parameters in surgical decisions. Patients with severe Class II skeletal malocclusion commonly present with proclined and everted lower lip, chin muscle tension, and lip incompetence [14]. These patients face particular functional risks from genioplasty. Standard genioplasty procedures can result in postoperative complications including lip incompetence, chin ptosis, and increased lower incisor exposure [28]. For patients with lip dysfunction, genioplasty poses particular functional risks. The created bony step defect causes soft tissue collapse, potentially compromising lip function further. Orthognathic surgery offers a fundamental solution by restoring the maxillomandibular se changes all affect the measurement accuracy of Li-Sbl and Lia-Pog relationship and lip closure function. For Class II patients with poor soft tissue conditions or lip incompetence, orthognathic surgery is strongly recommended over isolated genioplasty.

### Interactive model complementing existing measurements

For skeletal Class II patients with mandibular retrusion, cephalometric analysis has established an extensive evaluation system. Traditional hard tissue measurements such as ANB, SNB, and SNA quantify skeletal discrepancies. Dental compensation parameters including incisor mandibular plane angle (IMPA), lower incisor to NB line (L1-NB), and interincisal angle assess alveolar adaptation. Research shows that Class II patients often exhibit compensatory labial inclination of lower incisors. This manifests as increased IMPA, increased L1-NB, decreased interincisal angle, and increased overjet [29]. However, traditional hard tissue measurements cannot directly reflect the impact of these skeletal and dental changes on soft tissue appearance [30]. Existing soft tissue assessment methods also have limitations. E-line and S-line measure horizontal distances but cannot distinguish whether lower lip protrusion results from "overall anterior displacement" or "inclination change" [7]. The mentolabial angle assesses sulcus morphology but is jointly influenced by lower lip posture and chin prominence. This makes it impossible to independently determine which component primarily causes the abnormality [5]. Determining whether the problem originates from lip position or chin prominence is critical for deciding between isolated genioplasty and combined orthodontic-surgical approach. These existing methods cannot provide clear answers to this question.

The Li-Sbl angle addresses these limitations by serving as an integrative soft tissue indicator. Lower lip morphology is influenced by mandibular sagittal position, lower incisor inclination, and soft tissue characteristics [31]. The Li-Sbl angle reflects the relationship between mandibular retrusion severity as measured by SNB and lower incisor compensation degree as measured by IMPA. When lower incisors compensate with labial inclination, Li is pushed anteriorly, decreasing the Li-Sbl angle. Simultaneously, mandibular retrusion causes posterior displacement of Sbl, also decreasing the Li-Sbl angle. Thus, Li-Sbl integrates the combined influence of skeletal foundation and dental compensation on soft tissue appearance. This is precisely what traditional hard tissue measurements alone cannot directly present. Lohia et al. found that soft tissue cephalometric analysis considering soft tissue thickness and individual variation performed better in predicting surgical treatment needs than traditional hard tissue analysis [32]. This supports the necessity of direct soft tissue assessment.

While Li-Sbl reflects the integrated influence of skeletal position measured by SNB and dental compensation measured by IMPA, establishing precise numerical conversion algorithms between these traditional measurements and Li-Sbl angles presents significant challenges. Individual soft tissue thickness variation means that the same skeletal and dental configuration may manifest as different Li-Sbl angles in patients with varying labial soft tissue characteristics. Therefore, we recommend direct measurement of Li-Sbl from the lateral cephalogram or clinical profile photograph rather than relying on conversions from hard tissue measurements. This approach ensures accurate assessment of the actual soft tissue manifestation that determines aesthetic perception. Future prospective studies incorporating both traditional cephalometric parameters and Li-Sbl measurements across diverse patient populations would be valuable.

Previous studies have explored interactions among lower facial structures. Mazhari et al. reported that ideal mentolabial sulcus depth varies with facial type, ranging from 5−7 mm for normal and short faces to 6–8 mm for long faces [33]. Modarai et al. indicated that for individuals with prominent chin, anterior lower lip displacement may enhance profile attractiveness [34]. However, few studies have specifically examined how lower lip inclination affects aesthetic perception relative to chin position.

Building on Naini's proposal of the Lia-Pog' angle, this study systematically introduced Li-Sbl as an independent lower lip inclination parameter [16]. We found that lower lip inclination changes the optimal chin position. When Li-Sbl increases to 70°, optimal Lia-Pog' shifts anteriorly to 0–10°. When Li-Sbl decreases to 30°, optimal Lia-Pog' shifts posteriorly to 20–30°. However, this compensation has a threshold. When Li-Sbl ≤ 30°, aesthetic scores remain low (females < 5, males < 4) even with optimized chin position. This is consistent with Alshammari's study showing that retroclined profiles still struggle to achieve satisfactory aesthetics even at maximum lip protrusion [35]. This indicates that extreme skeletal differences exceed dental and soft tissue compensatory capacity.

However, complete assessment of profile aesthetics should not be limited to the lower face. Romsics pointed out that harmony between nose and chin size exceeds the importance of individual structures. Profile contour is fundamentally determined by the interrelationship of the nose-lip-chin triad [36]. Recent studies have confirmed that nasal deviation significantly affects profile aesthetic perception. Coordination of subnasale and chin anterior–posterior position is crucial for facial contour attractiveness [37]. This study focused on lip-chin interaction and did not include nasal parameters. Although the Li-Sbl × Lia-Pog' model effectively captures lower facial interaction, comprehensive profile assessment ideally requires consideration of the nose as a third component. Nasal morphology—including nasal dorsum height, nasal tip projection, and nasolabial angle—affects perception of lower facial structures [38].

### Population-specific considerations

Our results demonstrate that for Chinese evaluators, females achieve optimal aesthetic outcomes with Li-Sbl 45° and Lia-Pog' at 20°, while males show a wider acceptable range. This is consistent with existing studies on Asian populations. Lee et al. reported that Koreans prefer Lia-Pog' in the 0–25° range, similar to our findings for Chinese [6]. Studies involving Asian populations suggest a preference for mild chin retrusion [39] In contrast, Naini et al. reported that Caucasians prefer Lia-Pog' closer to 0° [16], reflecting greater acceptance of prominent chins in Western populations.

Gender differences in facial profile aesthetic perception are also evident. Males and females present different optimal chin positions. For the same lower lip inclination, males have smaller optimal Lia-Pog' angles with more prominent chins, while females prefer relatively retruded chin positions. Previous studies similarly reported that ideal female mandibular profile is more retruded than ideal male profile, with prominence considered a masculine feature [2, 18]. In female profiles, only the FL45C20 configuration received significantly higher scores than other configurations (*P* < 0.05), indicating a narrower optimal range. In contrast, males had a wider ideal aesthetic range (ML45C10-20 and ML70C0). This is consistent with Bertot's finding that male facial profiles have wider aesthetic acceptability than female profiles [40]. This may reflect narrower tolerance for structural deviation in female faces. Therefore, aesthetic standards and treatment goals should consider gender-specific differences, avoiding unified standards that mask gender distinctions.

This study investigated differences in aesthetic perception among four evaluator groups: maxillofacial surgeons, orthodontists, skeletal Class II patients, and laypeople. Results showed high consistency among groups for facial configurations approaching ideal proportions, consistent with previous studies showing shared aesthetic perception baselines [16, 41]. This consensus on ideal configurations is crucial for establishing clinical treatment goals. However, significant inter-group differences emerged when configurations significantly deviated from ideal.

For Li-Sbl 30° configurations, evaluator group differences were most significant. Orthodontists showed the highest tolerance for severe lower lip inclination. Class II patients scored significantly lower than orthodontists (mean difference 0.58–1.58 points; *r* = 0.29–0.43) and laypeople (mean difference 0.59–1.26 points; *r* = 0.25–0.37), confirming heightened sensitivity to mandibular retrusion-related features. Maxillofacial surgeons' ratings were intermediate. This is consistent with findings that orthognathic patients show higher sensitivity to their own facial defects [42].

Surgical recommendations revealed systematic divergence among evaluator groups in clinical decision-making. Naini proposed a surgical threshold for chin retrusion at a Lia-Pog' angle of 25° [16], while Lee suggested a larger threshold angle of 40° [6]. However, these early studies primarily used silhouettes and did not consider Li-Sbl angle as a key variable. Our study reveals that while Lia-Pog' 40° achieved consensus for surgical intervention across groups, surgical propensity at other Lia-Pog' angles varied substantially across Li-Sbl configurations.

For configurations clearly requiring surgical intervention (*n* = 13), all groups demonstrated high recommendation rates: surgeons 85.7%, patients 83.2%, orthodontists 79.0%, and laypeople 69.8%. For configurations not requiring surgery (*n* = 8), all groups showed low recommendation rates: surgeons 10.0%, orthodontists 1.1%, laypeople 11.8%, and patients 15.6%. These patterns indicate interprofessional consensus when clinical necessity is unambiguous. However, in surgical borderline configurations (*n* = 7), inter-group differences intensified dramatically (mean Cramér's V = 0.306, *P* < 0.008). Surgeons maintained recommendations at 54.0% and patients at 57.6%, while orthodontists' rate dropped to 25.7%. Laypeople showed an intermediate pattern at 44.1%. This demonstrates that orthodontists become markedly conservative when treatment necessity is uncertain, consistent with interprofessional differences reported by Voon et al. [43].

These patterns reveal a fundamental distinction between aesthetic perception and treatment need. Configurations with extremely low aesthetic scores (Li-Sbl 30°) elicited significant inter-group differences in aesthetic perception. However, they achieved consensus on treatment necessity. The opposite occurred for borderline configurations with moderate aesthetic scores. These showed similar aesthetic perceptions but divergent surgical recommendations. Aesthetic perception evaluates the degree of facial attractiveness. Treatment need determines whether intervention is warranted. It integrates not only perceived aesthetic deficit, but also the nature of deformity, improvement potential, surgical complexity, and risk–benefit ratios. Thus, treatment decisions cannot be determined solely by aesthetic scores. Multidisciplinary consultation is essential for borderline cases.

Evaluator age and gender had minimal impact on aesthetic scores and surgical recommendations. Effect sizes were far smaller than professional background differences. This is consistent with previous studies indicating that core standards of facial aesthetic perception have relative stability across different age groups and genders [8, 44].

### Methodological strengths and limitations

The core methodological innovation of this study was establishing two truly independent and clinically modifiable aesthetic parameters. Naini previously proposed decomposing the mentolabial angle into upper and lower components, but geometric analysis shows these angles are not completely independent as the Sbl point moves with lower lip movement [45]. This study selected the Lia-Pog' angle validated by Naini et al., combining it with Li-Sbl to form a two-parameter model [16]. The key advantage lies in parameter independence: Li-Sbl independently quantifies lower lip inclination, comprehensively reflecting mandibular position, lower incisor inclination, and soft tissue morphology, modifiable through orthodontic-orthognathic treatment. Lia-Pog' independently quantifies chin prominence, directly adjustable through genioplasty. This correspondence between parameter independence and surgical modifiability enables clinicians to determine whether aesthetic problems originate from lower lip inclination, chin prominence, or both.

A second strength is the diversified evaluator design. We included maxillofacial surgeons, orthodontists, skeletal Class II patients, and laypeople. As the primary clinical population for this model, Class II patients' perspectives have decisive significance for evaluating treatment effects. Including two professional groups captures inherent multidisciplinary divergence in orthognathic decision-making. This design provides empirical evidence for multidisciplinary consultation and patient communication in clinical practice.

A third methodological strength is the use of 2D profile analysis, which is the appropriate method for establishing the fundamental relationship between lower lip inclination and chin prominence. Li-Sbl and Lia-Pog' are sagittal plane parameters with aesthetic effects manifesting primarily in profile view. 2D analysis allows precise control and systematic variation of these parameters while holding other facial dimensions constant. This methodological control is essential for isolating their specific interactive effects, which would be confounded by additional variables in 3D assessment (such as facial width, symmetry, and rotational angles). Furthermore, 2D profile analysis maintains direct clinical relevance. Lateral cephalometric measurement remains the standard for orthognathic surgical planning, and previous validation studies have confirmed the reliability of 2D profile images for assessing sagittal parameters [18, 35]. The direct correspondence between Li-Sbl × Lia-Pog' and standard cephalometric measurements facilitates immediate clinical application.

This study has several limitations. First, the use of 2D profile images cannot present frontal view information such as chin width and facial symmetry. Real facial aesthetic perception involves dynamic expressions, multi-angle observation, and 3D morphology. These frontal and 3D features may affect overall evaluation of facial perception and surgical decisions, limiting direct translation of results to 3D clinical assessment. Second, only one model per gender was used as the base template. This design choice was necessary to achieve experimental control, eliminating confounding effects of facial morphology variation. It isolated the specific effects of Li-Sbl and Lia-Pog' changes. However, each template represents a specific combination of facial type, ethnic characteristics, and nasal morphology. This limits generalizability across diverse populations. Nasal morphology is a particularly important confounding variable that can substantially alter the perceived balance between lower lip and chin. This may affect the optimal Li-Sbl and Lia-Pog' values in patients with atypical nasal features. Third, this study evaluated female images first, then male images, potentially causing male image evaluation to be affected by fatigue effects and aesthetic baseline establishment from previous assessments. To reduce bias, all 30 images were simultaneously presented before formal scoring, allowing evaluators to form preliminary impressions of aesthetic difference ranges. Male and female images were analyzed separately as independent datasets without cross-gender comparisons. Nevertheless, we cannot completely rule out potential influences of fatigue effects or standard drift. Fourth, due to privacy-focused consent procedures, we could not systematically document complete cephalometric records and orthodontic treatment stage for all patient evaluators, though all had confirmed skeletal Class II diagnoses and no history of surgical intervention.

### Future research directions

Future research can extend this study's findings in several directions. 3D validation studies combining CBCT and 3D facial scanning can simultaneously acquire sagittal parameters (Li-Sbl and Lia-Pog') and frontal parameters (e.g., chin width, facial symmetry, mandibular width) to validate the model's applicability in 3D space, with dynamic video assessment further capturing the influence of expressions and movements on aesthetic perception. Population diversity should be expanded to include Chinese individuals with varied facial morphologies (e.g., lip thickness, facial proportions) and other ethnic groups, exploring how baseline facial characteristics and ethnicity influence lower lip-chin aesthetic standards. The assessment scope should extend from the lower face to the nose-lip-chin triad, incorporating nasal parameters such as dorsal height, tip projection, and nasolabial angle. Regarding evaluator characterization, future studies should systematically document patient evaluators' complete skeletal measurements, treatment history, and treatment stage to better understand how individual facial characteristics and orthodontic progress influence aesthetic perception. Prospective clinical studies should track pre- and postoperative Li-Sbl and Lia-Pog' changes in patients undergoing orthognathic surgery or genioplasty, while systematically collecting patient-reported outcomes using standardized instruments (FACE-Q, OQLQ) to assess facial satisfaction, psychosocial well-being, and quality of life, thereby validating the model's clinical predictive value and correlating objective measurements with subjective patient outcomes.

## Conclusions

This study establishes the Li-Sbl × Lia-Pog' model as a quantitative framework for lower facial aesthetic assessment and orthognathic treatment planning. Significant Li-Sbl × Lia-Pog' interactions revealed a compensation mechanism. As Li-Sbl decreases, the optimal Lia-Pog' angle increases, indicating less chin advancement is required. However, this compensation is exceeded at Li-Sbl ≤ 30°, where even optimal chin positioning fails to achieve acceptable aesthetic outcomes. For the Chinese population, optimal configurations are gender-specific. Females achieve best results at Li-Sbl 45° with Lia-Pog' 20°, while males perform optimally at Li-Sbl 45° with Lia-Pog' 10°–20° or Li-Sbl 70° with Lia-Pog' 0°. Evaluator background significantly influences perception and surgical recommendations, with Class II patients rating Li-Sbl 30° profiles significantly lower than orthodontists and laypeople. For borderline configurations, maxillofacial surgeons and patients favor intervention while orthodontists adopt conservative approaches.

For clinicians, Li-Sbl and Lia-Pog' should be routine measurements, and the clinical decision matrix provided allows rapid treatment classification. For borderline cases, clinicians should anticipate divergent opinions and seek collaborative decision-making. Critically, when Li-Sbl ≤ 30°, isolated genioplasty is insufficient and comprehensive orthognathic surgery should be performed; when Li-Sbl > 30°, genioplasty is feasible with chin advancement determined by the compensation mechanism.

## Supplementary Information

Supplementary Material 1.

## Data Availability

The datasets used and/or analyzed during the current study are available from the corresponding author upon reasonable request.

## Abbreviations

2D: Two-Dimensional
3D: Three-Dimensional
ANB: A Point-Nasion-B Point Angle
CI: Confidence Interval
ICC: Intraclass Correlation Coefficient
IMPA: Incisor Mandibular Plane Angle
Li: Labrale Inferius
Lia: Labrale Inferius Anteriors
Li-Sbl: Lower Lip Inclination Angle
Lia-Pog': Lower Lip-Chin Prominence Angle
NRS: Numerical Rating Scale
Pog': Soft-Tissue Pogonion
Sbl: Sublabiale
SD: Standard Deviation
SNB: Sella-Nasion-B Point Angle
TrH: True Horizontal Line
TrV: True Vertical Line

## References

[1] Nongthombam H, Kumar M, Goyal M, Abrar M, Shaha KS, Kumar S (2023) Regional influence on the aesthetic preference of different Mongolian profiles: A comparative study of assessors from Northeast and Mainland India. Int Orthod 21(2):100730. 10.1016/j.ortho.2023.100730 36773557 10.1016/j.ortho.2023.100730

[2] Doan LL, Ives GC, Cordero JJ, Lee JC (2024) A differential analysis of preferred feminine facial contours for transfeminine individuals. J Craniofac Surg 35(5):1389–1393. 10.1097/SCS.0000000000010238 38738872 10.1097/SCS.0000000000010238

[3] Hsieh DM, He H, Zhong S, Liew S, Wu Y (2022) Chin microgenia: An anthropometric analysis on the prevalence and severity in a Chinese population. Dermatol Surg 48(5):516–522. 10.1097/DSS.0000000000003383 35125439 10.1097/DSS.0000000000003383

[4] Tipyanggul W, Changsiripun C, Chamnannidiadha N (2025) A comparison of esthetic preferences on female skeletal class II alterations among laypeople of different facial profiles. Eur J Dent 19(02):366–373. 10.1055/s-0044-1788654 39074835 10.1055/s-0044-1788654 PMC12020620

[5] Naini FB, Cobourne MT, Garagiola U, McDonald F, Wertheim D (2017) Mentolabial angle and aesthetics: a quantitative investigation of idealized and normative values. Max Plast Reconstr Surg 39(1):4. 10.1186/s40902-017-0102-8 10.1186/s40902-017-0102-8 PMC5292106 28217687

[6] Lee J, Han SH, Choi Y, Kim J, Park S, Kim Y (2022) Effect of observer’s sex and chin prominences on the perception of the lower lip-chin prominence angle. J Craniofac Surg 33(2):620–623. 10.1097/SCS.0000000000008138 34519713 10.1097/SCS.0000000000008138

[7] Golfeshan F, NasseriMojarad A, Sardarian AR (2024) Assessment of the acceptable range of lips and chin position in two different geographical zones of Iran among laypersons. J Dent (Shiraz) 25(2):169–177. 10.30476/dentjods.2023.97251.2003 38962081 10.30476/dentjods.2023.97251.2003 PMC11217067

[8] Adamek A, Sarul M, Lis J, Kobiela Z, Kiełczawa M, Semeniuk F (2022) Influence of lip projection and chin position on facial profile preferences among various layers of Polish population. Part 1. Clin Cosmet Investig Dent 14:253–263. 10.2147/CCIDE.S358452 36093269 10.2147/CCIDE.S358452 PMC9462944

[9] Santori F, Masedu F, Ciavarella D, Staderini E, Chimenti C, Tepedino M (2021) Effect of class II functional treatment on facial attractiveness, as perceived by professionals and laypeople. Sci Rep-UK 11(1):13989. 10.1038/s41598-021-93343-0 10.1038/s41598-021-93343-0 PMC8263773 34234201

[10] Dong T, Ye N, Yuan L, Wu S, Xia L, Fang B (2020) Assessing the influence of chin asymmetry on perceived facial esthetics with 3-dimensional images. J Oral Maxillofac Surg 78(8):1389–1396. 10.1016/j.joms.2020.03.017 32304663 10.1016/j.joms.2020.03.017

[11] Fadel R, Nicot R, Schlund M, Ferri J (2023) Simultaneous mandibular anterior segmental osteotomy and genioplasty: a novel technique. J Craniofac Surg 34(3):1064–1066. 10.1097/SCS.0000000000009049 36190696 10.1097/SCS.0000000000009049

[12] Keyhan SO, Cheshmi B, Fallahi HR, Asayesh MA, Fattahi T (2019) Balcony genioplasty: a novel technique for better esthetic results in patients with deep mentolabial fold. Maxillofac Plast Reconstr Surg 41(1):7. 10.1186/s40902-019-0190-8 30828571 10.1186/s40902-019-0190-8 PMC6369232

[13] Souza DBD, Oliveira AI, Gouvêa GR, Santamaria-Jr M (2022) What do black patients expect from orthodontic treatment? The aesthetic perception of facial profile between orthodontists and black laypersons. Dent Press J Orthod 27(4):e2220519. 10.1590/2177-6709.27.4.e2220519.oar 10.1590/2177-6709.27.4.e2220519.oar PMC9439571 36074432

[14] Perović T (2017) The influence of class II division 2 malocclusions on the harmony of the human face profile. Med Sci Monit 23:5589–5598. 10.12659/MSM.905453 29170363 10.12659/MSM.905453 PMC5712519

[15] Psomiadis S, Gkantidis N, Sifakakis I, Iatrou I (2024) Perceived effects of orthognathic surgery versus orthodontic camouflage treatment of convex facial profile patients. J Clin Med 13(1):91. 10.3390/jcm13010091 10.3390/jcm13010091 PMC10780077 38202096

[16] Naini FB, Garagiola U, Wertheim D (2019) Analysing chin prominence in relation to the lower lip: the lower lip-chin prominence angle. J Craniomaxillofac Surg 47(8):1310–1316. 10.1016/j.jcms.2019.06.002 31331858 10.1016/j.jcms.2019.06.002

[17] Moradinejad M, Rekabi A, Ashtiani AH, Atashkar N, Rakhshan V (2022) Psychometric and perceptometric comparisons of the perspectives of orthodontists, oral and maxillofacial surgeons, and laypeople of different ages and sexes towards beauty of female jaw angles (intergonial widths and gonial heights) on frontal and three‐quarter views. Biomed Res Int 2022(1):2595662. 10.1155/2022/2595662 36398071 10.1155/2022/2595662 PMC9666021

[18] Kucuk E, Yildirim M, Erdur EA (2025) Evaluation of the impact of mandibular symphysis region alterations on profile aesthetics. Sci Rep-UK 15(1):14832. 10.1038/s41598-025-99352-7 10.1038/s41598-025-99352-7 PMC12038029 40295733

[19] Emanuelli E, O’Connor MK, Garg RK (2023) Genioglossus advancement: technique modification for improved chin contour. PRS-Glob Open 11(3):e4846. 10.1097/GOX.0000000000004846 10.1097/GOX.0000000000004846 PMC9995103 36910733

[20] Naini FB, Gill DS. Principles of orthognathic treatment planning orthognathic surgery. 2016:170–213. 10.1002/9781119004370.ch6.

[21] Arriola-Guillén L, Díaz-Quevedo A, Rodríguez-Cárdenas Y, Ruíz-Mora G, Silveira HD (2025) Cephalometric features associated with the mentolabial angle and lower lip eversion in young adults: A cross-sectional study. J Clin Exp Dent. 17(8):e974–e979. 10.4317/jced.63016 40950515 10.4317/jced.63016 PMC12424598

[22] Fang ML, Choi SH, Choi YJ, Lee KJ (2023) Pattern of lip retraction according to the presence of lip incompetence in patients with Class II malocclusion. Korean J Orthod. 53(4):276–285. 10.4041/kjod22.260 37497584 10.4041/kjod22.260 PMC10387428

[23] Choi BK, Jeon HB, Lo LJ, Yun IS (2023) A retrospective analysis of redo orthognathic surgery: Underlying causes, strategy, and outcome. J Cranio Maxill Surg. 51(3):188–198. 10.1016/j.jcms.2023.01.018 10.1016/j.jcms.2023.01.018 36804362

[24] Şimşek B, Efeoglu C, ÖzdenYüce M, Akay MC, Çelen S (2021) Biomechanical validation of a modified genioplasty distractor. J Stomatol Oral Maxillofac Surg. 122(4):e33–e37. 10.1016/j.jormas.2021.03.001 33706028 10.1016/j.jormas.2021.03.001

[25] Singh G, Sharma S, Vignesh U, Katrolia R (2023) Combination of double-sliding advancement genioplasty and prearthroplastic distraction osteogenesis in cases of TMJ ankylosis with severe mandibular atrophy. Natl J Maxillofac Surg. 14(1):143–146. 10.4103/njms.njms_310_21 37273424 10.4103/njms.njms_310_21 PMC10235746

[26] Fariña R, Valladares-Pérez S, Navarro-Cuellar C, Torrealba R, Fariña-Silva A, Fariña-Silva G (2023) M-shaped Genioplasty: New Findings after 10 Years of Experience. PRS-GLOB OPEN. 11(1):e4778. 10.1097/GOX.0000000000004778 10.1097/GOX.0000000000004778 PMC9870213 36699236

[27] Kumar M, Singh RS, Singh G, Raj P, Gupta H, Kasrija R (2023) Hard and Soft Tissue Relapse After Different Genioplasty Procedures: A Scoping Review. Cureus J Med Sci. 15(7):e41478. 10.7759/cureus.41478 10.7759/cureus.41478 PMC10404160 37551245

[28] Saleh E, Saleh J, Saleh B (2021) Intraoral genioplasty-a newer technique. PRS-GLOB OPEN 9(4):e3518. 10.1097/GOX.0000000000003518 10.1097/GOX.0000000000003518 PMC8032354 33854863

[29] Laganà G, Malara A, Lione R, Bollero P, Cozza P (2025) Effects of class II elastics on lower incisors during treatment with clear aligners vs fixed appliance: a randomized clinical trial. Front Dent Med. 6:1613037. 10.3389/fdmed.2025.1613037 40708614 10.3389/fdmed.2025.1613037 PMC12286962

[30] Zhang C, Lu T, Wang L, Wen J, Huang Z, Lin S, Zhou Y, Li G, Li H (2024) Three-dimensional analysis of hard and soft tissue changes in skeletal class II patients with high mandibular plane angle undergoing surgery. Sci Rep-UK 14(1):2519. 10.1038/s41598-024-51322-1 10.1038/s41598-024-51322-1 PMC10827781 38291067

[31] Fioravanti KS, Mengoa MGR, Paludetto LV, Murayama GYA, Oliveira TM, Sforza C, Neppelenbroek KH, Soares S (2025) Comparative analysis of lip morphology in Brazilian caucasian individuals between 20 and 50 years old using stereophotogrammetry. Oral Maxillofac Surg 29(1):57. 10.1007/s10006-025-01349-z 39964586 10.1007/s10006-025-01349-z

[32] Lohia A, Shetty S, Singh A, Shetty S, M V A (2025) Evaluation of the level of agreement between clinical diagnosis and two cephalometric analyses: Cephalometric analysis for orthognathic surgery (COGS) and soft tissue cephalometric analysis (STCA). INT J DENT 2025:8655040. 10.1155/ijod/8655040 40125093 10.1155/ijod/8655040 PMC11928218

[33] Mazhari M, Rekabi A, Atashkar N, Khayami Z (2025) Ideal mentolabial sulcus depth in long-face, short-face, and normal-face in female individuals. Oral Maxillofac Surg 29(1):33. 10.1007/s10006-025-01336-4 39808315 10.1007/s10006-025-01336-4

[34] Modarai F, Donaldson JC, Naini FB (2013) The influence of lower lip position on the perceived attractiveness of chin prominence. ANGLE ORTHOD 83(5):795–800. 10.2319/122912-974.1 23530543 10.2319/122912-974.1 PMC8744523

[35] Alshammari AK, Algharbi MA, Alshammari SK, Alenzi AA, Malik YR, Abideen MZ, Siddiqui AA, Madfa AA (2023) Influence of lip position on esthetics perception with respect to profile divergence using silhouette images. BMC Oral Health 23(1):791. 10.1186/s12903-023-03537-3 37875850 10.1186/s12903-023-03537-3 PMC10598988

[36] Romsics L, Segatto A, Boa K, Becsei R, Rózsa N, Párkányi L, Pinke I, Piffkó J, Segatto E (2021) Patterns of facial profile preference in a large sample of dental students: a cross-sectional study. Int J Environ Res Public Health 18(16):8554. 10.3390/ijerph18168554 34444300 10.3390/ijerph18168554 PMC8394490

[37] Chen T, Yang X, Xue C, Bai D, Xu H (2025) Harmonizing soft tissue subnasale and chin position in a forehead-based framework: interracial commonalities and differences between Asian and Caucasian females. Angle Orthod 95(1):86–95 39317377 10.2319/022524-145.1 PMC11662358

[38] Patel PN, Most SP (2020) Concepts of facial aesthetics when considering ethnic rhinoplasty. Otolaryngol Clin North Am 53(2):195–208 32008729 10.1016/j.otc.2019.12.001

[39] Tan SK, Leung WK, Tang ATH, Zwahlen RA (2022) Facial profile study using 3-dimensional photographs to assess esthetic preferences of Hong Kong Chinese orthognathic patients and laypersons. Am J Orthod Dentofacial Orthop 161(2):e105–e113. 10.1016/j.ajodo.2021.01.024 34531091 10.1016/j.ajodo.2021.01.024

[40] Bertot AA, Dammling CW, Souccar NM, Louis PJ, Zhai G, Kinard BE (2024) A cross-sectional study examining Andrews’ analysis in Caucasian and African American subjects. J Oral Maxillofac Surg 82(12):1528–1536. 10.1016/j.joms.2024.08.059 39278262 10.1016/j.joms.2024.08.059 PMC11611624

[41] Imani MM, Sanei E, Niaki EA, Shahroudi AS (2018) Esthetic preferences of orthodontists, oral surgeons, and laypersons for Persian facial profiles. Am J Orthod Dentofacial Orthop 154(3):412–420. 10.1016/j.ajodo.2017.11.040 30173845 10.1016/j.ajodo.2017.11.040

[42] Naini FB, Laskin DM, Garagiola U, Cobourne MT, McDonald F, Wertheim D (2020) The opinion of different observer groups about the esthetic impact and need for surgical correction of varying submental lengths. J Oral Maxillofac Surg 78(4):630–631. 10.1016/j.joms.2019.11.023 10.1016/j.joms.2019.11.023 31881172

[43] Voon KKR, Lim AAT, Wong HC, Sim YF, Foong KWC (2023) Decision-making patterns among expert and novice orthodontists and oral maxillofacial surgeons in the management of adults with Class III malocclusions and moderate degree of skeletal discrepancies. J Orthod 50(4):410–422. 10.1177/14653125231181603 37357426 10.1177/14653125231181603

[44] Yabe A, Ikoma M, Arai K (2021) Evaluations of the facial attractiveness of young women with severe maxillary anterior crowding by orthodontists and laypeople with and without orthodontic treatment experience in Japan. Am J Orthod Dentofacial Orthop 159(6):750–757. 10.1016/j.ajodo.2020.02.017 33888377 10.1016/j.ajodo.2020.02.017

[45] Naini FB. Facial aesthetics: concepts and clinical diagnosis. John Wiley & Sons. 2011.

