# Supplementary material for: Lower lip inclination and chin prominence: interactive effects on facial aesthetics and implications for orthognathic treatment planning
Source: Maxillofac Plast Reconstr Surg. 2026 Apr 6;48(1):8. doi: 10.1186/s40902-026-00501-3 (PMC13129129; doi:10.1186/s40902-026-00501-3)
Supplement: Supplementary file 1 — Supplementary Material 1. [file 40902_2026_501_MOESM1_ESM.zip › Supplementary Material 1.docx]

**Clinical Documentation Template**

For use with Li-Sbl × Lia-Pog' Clinical Decision Support System

# PATIENT INFORMATION

| Patient Name |  | Medical Record No. |  |
| --- | --- | --- | --- |
| Gender | ☐ Female ☐ Male | Age |  |
| Assessment Date |  |  |  |

# CLINICAL MEASUREMENTS

| **Parameter** | **Measurement** | **Notes** |
| --- | --- | --- |
| **Li-Sbl angle (°)** |  |  |
| **Lia-Pog' angle (°)** |  |  |
| SNB angle (°) |  |  |
| IMPA (°) |  |  |
| ANB angle (°) |  |  |

# MATRIX CONSULTATION RESULTS

| **Aesthetic Assessment** | **Results** |
| --- | --- |
| Mean aesthetic score (0-10) |  |
| Surgical recommendation rate | Range: _____ to _____ (%) |
| **Clinical classification** | ☐ Surgery Not Indicated (Green) ☐ Surgery Borderline (Yellow) ☐ Surgery Indicated (Red) |

**Reference: Aesthetic Standards for Li-Sbl and Lia-Pog' Angles (Chinese Population)**

| **Classification** | **Female** | **Male** |
| --- | --- | --- |
| **Optimal** | Li-Sbl 45° + Lia-Pog' 20° (score 7.99) | Li-Sbl 45° + Lia-Pog' 10°-20° (scores 6.94-7.08) or Li-Sbl 70° + Lia-Pog' 0° (score 6.60) |
| **Surgery Not Indicated** | Li-Sbl 45° + Lia-Pog' 10° (score 6.59) Li-Sbl 70° + Lia-Pog' 0°-20° (scores 4.45-5.85) | Li-Sbl 70° + Lia-Pog' 10° (score 5.97) |

*Note: Aesthetic scores <4.7 typically indicate surgery required; borderline cases require individualized assessment.*

# IMPROVEMENT POTENTIAL

| Isolated genioplasty | + _____ points |
| --- | --- |
| Orthognathic surgery | + _____ points |
| **Difference (OS - G)** | + _____ points |

# FUNCTIONAL ASSESSMENT

| Lip competence | ☐ Competent ☐ Incompetent |
| --- | --- |
| Malocclusion | ☐ None ☐ Class I ☐ Class II ☐ Class III |
| TMJ disorder | ☐ No ☐ Yes (specify): __________ |
| OSA symptoms | ☐ No ☐ Yes |

# TREATMENT DECISION

**Recommended treatment approach:**

☐ Orthodontic/camouflage treatment only

☐ Isolated genioplasty (target Lia-Pog': _____°)

☐ Orthognathic surgery

Target Li-Sbl: _____° Target Lia-Pog': _____°

☐ Multidisciplinary consultation required (borderline case)

# CLINICAL NOTES AND RATIONALE

|  |
| --- |

|  |  |
| --- | --- |
| Clinician Signature | Date |

# POST-OPERATIVE ASSESSMENT

(To be completed at: ☐ 3 months ☐ 6 months ☐ 12 months ☐ Other: _____)

**Post-operative Measurements**

| **Parameter** | **Pre-op** | **Target** | **Post-op** | **Achieved** |
| --- | --- | --- | --- | --- |
| **Li-Sbl angle (°)** |  |  |  |  |
| **Lia-Pog' angle (°)** |  |  |  |  |
| SNB angle (°) |  |  |  |  |
| IMPA (°) |  |  |  |  |
| Aesthetic score (0-10) |  |  |  |  |

**Outcome Assessment**

| Target achievement | ☐ Complete ☐ Partial ☐ Not achieved |
| --- | --- |
| Functional improvement | ☐ Improved ☐ Unchanged ☐ Worsened |
| Complications | ☐ None ☐ Yes (specify): __________ |
| Patient satisfaction (1-10) |  |
| Overall outcome | ☐ Optimal ☐ Satisfactory ☐ Acceptable ☐ Suboptimal |

# Post-operative Notes

|  |
| --- |

|  |  |
| --- | --- |
| Clinician Signature | Date |
