# Supplementary material for: Lower lip inclination and chin prominence: interactive effects on facial aesthetics and implications for orthognathic treatment planning
Source: Maxillofac Plast Reconstr Surg. 2026 Apr 6;48(1):8. doi: 10.1186/s40902-026-00501-3 (PMC13129129; doi:10.1186/s40902-026-00501-3)
Supplement: Supplementary file 1 — Supplementary Material 1. [file 40902_2026_501_MOESM1_ESM.zip › Supplementary Tables.docx]

# Supplementary Tables

**Supplementary Table 1.** Pairwise Comparisons of Female Images Between Evaluator Groups (Significant Differences Only)

| **Image** | **Group 1** | **Mean ±SD** | **Group 2** | **Mean ±SD** | **r** | **P-value** |
| --- | --- | --- | --- | --- | --- | --- |
| FL30C0 | Orthodontist | 1.93±1.60 | Class II Patient | 0.98±1.18 | 0.292 | 0.002* |
| FL30C0 | Class II Patient | 0.98±1.18 | Laypeople | 1.70±1.36 | 0.284 | <0.001* |
| FL30C10 | Class II Patient | 1.71±1.54 | Laypeople | 2.58±1.48 | 0.312 | <0.001* |
| FL30C20 | Orthodontist | 3.04±1.48 | Class II Patient | 2.09±1.50 | 0.314 | 0.001* |
| FL30C20 | Class II Patient | 2.09±1.50 | Laypeople | 3.10±1.48 | 0.335 | <0.001* |
| FL30C30 | Class II Patient | 4.27±1.55 | Laypeople | 5.16±1.64 | 0.275 | <0.001* |
| FL30C40 | Class II Patient | 2.82±1.57 | Laypeople | 4.08±1.73 | 0.366 | <0.001* |
| FL45C30 | Maxillofacial Surgeon | 5.00±1.26 | Orthodontist | 5.80±1.07 | 0.348 | 0.004* |
| FL70C10 | Orthodontist | 6.27±0.83 | Class II Patient | 5.52±1.22 | 0.340 | <0.001* |
| FL70C30 | Orthodontist | 3.20±1.02 | Class II Patient | 2.40±1.12 | 0.297 | 0.002* |
| FL70C40 | Maxillofacial Surgeon | 1.49±1.56 | Laypeople | 2.23±1.37 | 0.270 | 0.008* |

Note. Values are mean ± SD. r = effect size (adjusted α = 0.008). *P < 0.05.

**Supplementary Table 2. Pairwise Comparisons of Female Images Within Each Evaluator Group**

Note. This table contains complete pairwise comparisons within each evaluator group (105 comparisons per group, 4 groups). The complete data is provided in a separate Excel file (Supplementary-Table-S3-Female.xlsx).

**Supplementary Table 3.** Pairwise Comparisons of Male Images Between Evaluator Groups (Significant Differences Only)

| **Image** | **Group 1** | **Mean ±SD** | **Group 2** | **Mean ±SD** | **r** | **P-value** |
| --- | --- | --- | --- | --- | --- | --- |
| ML30C0 | Orthodontist | 2.69±1.62 | Class II Patient | 1.44±1.43 | 0.350 | <0.001* |
| ML30C10 | Orthodontist | 3.38±1.60 | Class II Patient | 1.80±1.58 | 0.427 | <0.001* |
| ML30C10 | Class II Patient | 1.80±1.58 | Laypeople | 2.68±1.78 | 0.251 | 0.004* |
| ML30C20 | Orthodontist | 4.56±1.50 | Class II Patient | 3.37±1.73 | 0.321 | <0.001* |
| ML30C30 | Orthodontist | 3.84±1.25 | Class II Patient | 2.90±0.93 | 0.375 | <0.001* |
| ML30C30 | Class II Patient | 2.90±0.93 | Laypeople | 3.83±1.83 | 0.293 | <0.001* |
| ML30C40 | Maxillofacial Surgeon | 2.16±1.49 | Laypeople | 3.13±1.47 | 0.294 | 0.003* |
| ML30C40 | Class II Patient | 2.09±1.85 | Laypeople | 3.13±1.47 | 0.308 | <0.001* |
| ML45C0 | Maxillofacial Surgeon | 4.98±1.02 | Class II Patient | 3.91±1.00 | 0.351 | <0.001* |
| ML45C0 | Maxillofacial Surgeon | 4.98±1.02 | Laypeople | 4.02±0.98 | 0.323 | <0.001* |
| ML45C0 | Orthodontist | 5.38±0.88 | Class II Patient | 3.91±1.00 | 0.350 | <0.001* |
| ML45C0 | Orthodontist | 5.38±0.88 | Laypeople | 4.02±0.98 | 0.330 | <0.001* |
| ML45C10 | Orthodontist | 7.60±1.10 | Laypeople | 6.72±1.45 | 0.281 | 0.005* |
| ML45C40 | Maxillofacial Surgeon | 2.16±1.60 | Laypeople | 3.33±1.58 | 0.317 | 0.001* |

Note. Values are mean ± SD. r = effect size (adjusted α = 0.008). *P < 0.05.

**Supplementary Table 4. Pairwise Comparisons of Male Images Within Each Evaluator Group**

Note. This table contains complete pairwise comparisons within each evaluator group (105 comparisons per group, 4 groups). The complete data is provided in a separate Excel file (Supplementary-Table-S4-Male.xlsx).

**Supplementary Table 5.** Comparison of Attractiveness Scores for Female Images by Age Group

| **Image** | **Age<30(N=150)** | **Age≥30(N=120)** | **r** | **P value** |
| --- | --- | --- | --- | --- |
| FL30C0 | 1.31±1.38 | 1.57±1.30 | 0.137 | 0.111 |
| FL30C10 | 1.93±1.56 | 2.37±1.53 | 0.177 | 0.020* |
| FL30C20 | 2.41±1.56 | 3.08±1.50 | 0.246 | 0.004* |
| FL30C30 | 4.50±1.73 | 4.89±1.45 | 0.156 | 0.044* |
| FL30C40 | 3.21±1.74 | 3.68±1.62 | 0.152 | 0.022* |
| FL45C0 | 2.83±1.55 | 3.26±1.62 | 0.161 | 0.027* |
| FL45C10 | 6.54±1.30 | 6.65±1.23 | 0.042 | 0.479 |
| FL45C20 | 7.96±1.05 | 8.02±0.91 | 0.008 | 0.636 |
| FL45C30 | 5.43±1.10 | 5.41±1.23 | -0.031 | 0.862 |
| FL45C40 | 2.65±1.39 | 2.96±1.45 | 0.120 | 0.081 |
| FL70C0 | 5.84±1.24 | 5.87±1.22 | 0.009 | 0.859 |
| FL70C10 | 5.63±1.26 | 5.92±1.13 | 0.150 | 0.056 |
| FL70C20 | 4.37±1.09 | 4.56±1.10 | 0.129 | 0.154 |
| FL70C30 | 2.65±1.11 | 2.88±1.05 | 0.107 | 0.084 |
| FL70C40 | 1.87±1.44 | 1.86±1.31 | 0.020 | 0.929 |

Note. Values are mean ± SD. r = effect size. *P < 0.05.

**Supplementary Table 6.** Comparison of Attractiveness Scores for Male Images by Age Group

| **Image** | **Age<30(N=150)** | **Age≥30(N=120)** | **r** | **P value** |
| --- | --- | --- | --- | --- |
| ML30C0 | 1.77±1.54 | 2.35±1.81 | 0.173 | 0.006* |
| ML30C10 | 2.19±1.74 | 2.88±1.70 | 0.232 | 0.001* |
| ML30C20 | 3.60±2.03 | 4.26±1.78 | 0.219 | 0.005* |
| ML30C30 | 3.21±1.46 | 3.78±1.48 | 0.225 | 0.002* |
| ML30C40 | 2.44±1.69 | 2.58±1.68 | 0.049 | 0.514 |
| ML45C0 | 4.15±1.03 | 4.64±1.21 | 0.246 | ＜0.001* |
| ML45C10 | 7.13±1.18 | 7.02±1.28 | -0.063 | 0.469 |
| ML45C20 | 6.96±1.39 | 6.91±1.24 | -0.048 | 0.747 |
| ML45C30 | 3.53±1.89 | 3.96±1.65 | 0.129 | 0.053 |
| ML45C40 | 2.79±1.58 | 2.87±1.70 | 0.016 | 0.692 |
| ML70C0 | 6.53±1.79 | 6.68±1.58 | 0.031 | 0.467 |
| ML70C10 | 5.77±2.12 | 6.22±1.73 | 0.116 | 0.056 |
| ML70C20 | 4.64±1.78 | 4.78±1.52 | 0.060 | 0.503 |
| ML70C30 | 3.15±1.70 | 3.38±1.60 | 0.100 | 0.256 |
| ML70C40 | 1.99±1.62 | 2.12±1.52 | 0.064 | 0.493 |

Note. Values are mean ± SD. r = effect size. *P < 0.05.

**Supplementary Table 7.** Comparison of Female Image Attractiveness Scores by Evaluator Gender

| **Image** | **Female(N=167)** | **Male(N=103)** | **r** | **P value** |
| --- | --- | --- | --- | --- |
| FL30C0 | 1.43±1.41 | 1.43±1.25 | 0.024 | 0.981 |
| FL30C10 | 2.08±1.59 | 2.18±1.52 | 0.048 | 0.604 |
| FL30C20 | 2.61±1.61 | 2.87±1.51 | 0.078 | 0.175 |
| FL30C30 | 4.50±1.69 | 4.95±1.47 | 0.140 | 0.225 |
| FL30C40 | 3.37±1.71 | 3.50±1.69 | 0.043 | 0.531 |
| FL45C0 | 3.08±1.67 | 2.91±1.46 | -0.064 | 0.377 |
| FL45C10 | 6.50±1.23 | 6.74±1.33 | 0.125 | 0.138 |
| FL45C20 | 8.00±0.97 | 7.96±1.03 | -0.013 | 0.758 |
| FL45C30 | 5.49±1.13 | 5.31±1.19 | -0.096 | 0.219 |
| FL45C40 | 2.70±1.47 | 2.93±1.33 | 0.112 | 0.184 |
| FL70C0 | 5.84±1.25 | 5.86±1.20 | 0.010 | 0.897 |
| FL70C10 | 5.74±1.20 | 5.78±1.23 | 0.008 | 0.823 |
| FL70C20 | 4.49±1.05 | 4.40±1.17 | -0.029 | 0.539 |
| FL70C30 | 2.70±1.09 | 2.83±1.08 | 0.060 | 0.359 |
| FL70C40 | 1.91±1.44 | 1.80±1.29 | -0.030 | 0.501 |

Note. Values are mean ± SD. r = effect size. No significant differences (all P > 0.05).

**Supplementary Table 8.** Comparison of Male Image Attractiveness Scores by Evaluator Gender

| **Image** | **Female(N=167)** | **Male(N=103)** | **r** | **P value** |
| --- | --- | --- | --- | --- |
| ML30C0 | 2.05±1.72 | 1.99±1.64 | 0.007 | 0.761 |
| ML30C10 | 2.47±1.79 | 2.54±1.70 | -0.043 | 0.746 |
| ML30C20 | 3.89±2.02 | 3.89±1.83 | 0.054 | 0.997 |
| ML30C30 | 3.41±1.45 | 3.54±1.56 | -0.001 | 0.495 |
| ML30C40 | 2.37±1.68 | 2.72±1.68 | -0.037 | 0.095 |
| ML45C0 | 4.37±1.14 | 4.37±1.14 | -0.027 | 0.987 |
| ML45C10 | 6.98±1.22 | 7.23±1.22 | -0.091 | 0.102 |
| ML45C20 | 7.04±1.33 | 6.78±1.31 | 0.003 | 0.117 |
| ML45C30 | 3.62±1.87 | 3.88±1.66 | -0.011 | 0.224 |
| ML45C40 | 2.72±1.64 | 2.99±1.62 | -0.013 | 0.183 |
| ML70C0 | 6.54±1.70 | 6.70±1.71 | 0.044 | 0.455 |
| ML70C10 | 5.89±2.06 | 6.09±1.80 | 0.044 | 0.414 |
| ML70C20 | 4.63±1.79 | 4.81±1.46 | 0.059 | 0.392 |
| ML70C30 | 3.17±1.77 | 3.40±1.47 | 0.087 | 0.248 |
| ML70C40 | 2.08±1.68 | 2.01±1.39 | 0.010 | 0.719 |

Note. Values are mean ± SD. r = effect size. No significant differences (all P > 0.05).

.

**Supplementary Table 9.** Comparison of Surgical Recommendation Rates for Female Images by Age Group

| **Image** | **Age<30(N=150)** | **Age≥30(N=120)** | **Φ** | **OR** | **95% CI** | **P value** |
| --- | --- | --- | --- | --- | --- | --- |
| FL30C0 | 88.7% | 95.8% | 0.117 | 0.34 | [0.12, 0.95] | 0.056 |
| FL30C10 | 90.7% | 81.7% | 0.121 | 2.18 | [1.06, 4.47] | 0.048* |
| FL30C20 | 82.0% | 66.7% | 0.168 | 2.28 | [1.30, 4.00] | 0.006* |
| FL30C30 | 58.0% | 46.0% | 0.114 | 1.63 | [1.01, 2.65] | 0.062 |
| FL30C40 | 78.7% | 71.7% | 0.072 | 1.46 | [0.84, 2.54] | 0.235 |
| FL45C0 | 76.0% | 70.8% | 0.050 | 1.30 | [0.76, 2.25] | 0.413 |
| FL45C10 | 12.0% | 9.2% | 0.033 | 1.35 | [0.61, 2.98] | 0.583 |
| FL45C20 | 4.7% | 3.3% | 0.015 | 1.42 | [0.41, 4.97] | 0.810 |
| FL45C30 | 30.7% | 37.5% | 0.064 | 0.74 | [0.44, 1.22] | 0.293 |
| FL45C40 | 83.3% | 84.2% | 0.001 | 0.94 | [0.49, 1.80] | 0.985 |
| FL70C0 | 12.7% | 11.7% | 0.004 | 1.10 | [0.53, 2.29] | 0.950 |
| FL70C10 | 18.0% | 15.0% | 0.030 | 1.24 | [0.65, 2.39] | 0.622 |
| FL70C20 | 34.7% | 37.5% | 0.022 | 0.88 | [0.54, 1.46] | 0.723 |
| FL70C30 | 78.7% | 75.0% | 0.034 | 1.23 | [0.70, 2.17] | 0.571 |
| FL70C40 | 96.7% | 94.2% | 0.042 | 1.80 | [0.56, 5.81] | 0.488 |

Note. Values are percentages. φ = phi; OR = odds ratio. *P < 0.05.

**Supplementary Table 10.** Comparison of Surgical Recommendation Rates for Male Images by Age Group

| **Image** | **Age<30(N=150)** | **Age≥30(N=120)** | **Φ** | **OR** | **95% CI** | **P value** |
| --- | --- | --- | --- | --- | --- | --- |
| ML30C0 | 90.7% | 90.8% | 0.000 | 0.98 | [0.43, 2.25] | 1.000 |
| ML30C10 | 88.7% | 81.7% | 0.088 | 1.76 | [0.89, 3.48] | 0.147 |
| ML30C20 | 56.0% | 45.0% | 0.102 | 1.56 | [0.96, 2.52] | 0.094 |
| ML30C30 | 64.7% | 60.8% | 0.032 | 1.18 | [0.72, 1.94] | 0.602 |
| ML30C40 | 68.0% | 70.8% | 0.022 | 0.88 | [0.52, 1.47] | 0.712 |
| ML45C0 | 51.3% | 40.8% | 0.097 | 1.53 | [0.94, 2.48] | 0.111 |
| ML45C10 | 13.3% | 6.7% | 0.096 | 2.15 | [0.91, 5.08] | 0.113 |
| ML45C20 | 8.7% | 5.0% | 0.057 | 1.80 | [0.66, 4.89] | 0.352 |
| ML45C30 | 57.3% | 51.7% | 0.049 | 1.26 | [0.78, 2.04] | 0.420 |
| ML45C40 | 77.3% | 81.7% | 0.044 | 0.77 | [0.42, 1.40] | 0.471 |
| ML70C0 | 10.7% | 7.5% | 0.041 | 1.47 | [0.63, 3.46] | 0.496 |
| ML70C10 | 18.7% | 11.7% | 0.086 | 1.74 | [0.87, 3.47] | 0.159 |
| ML70C20 | 32.7% | 39.2% | 0.060 | 0.75 | [0.46, 1.24] | 0.327 |
| ML70C30 | 66.0% | 72.5% | 0.062 | 0.74 | [0.44, 1.24] | 0.311 |
| ML70C40 | 90.0% | 92.5% | 0.031 | 0.73 | [0.31, 1.73] | 0.616 |

Note. Values are percentages. φ = phi; OR = odds ratio. *P < 0.05.

**Supplementary Table 11.** Comparison of Surgical Recommendation Rates for Female Images by Evaluator Gender

| **Image** | **Female(N=167)** | **Male(N=103)** | **Φ** | **OR** | **95% CI** | **P value** |
| --- | --- | --- | --- | --- | --- | --- |
| FL30C0 | 90.4% | 94.2% | 0.053 | 0.58 | [0.22, 1.54] | 0.386 |
| FL30C10 | 85.6% | 88.3% | 0.028 | 0.79 | [0.37, 1.65] | 0.649 |
| FL30C20 | 79.6% | 68.0% | 0.123 | 1.84 | [1.05, 3.23] | 0.054 |
| FL30C30 | 54.5% | 37.9% | 0.154 | 1.96 | [1.19, 3.24] | 0.011* |
| FL30C40 | 76.6% | 73.8% | 0.023 | 1.17 | [0.66, 2.06] | 0.700 |
| FL45C0 | 70.1% | 79.6% | 0.097 | 0.60 | [0.33, 1.07] | 0.112 |
| FL45C10 | 16.2% | 15.5% | 0.000 | 1.05 | [0.53, 2.06] | 1.000 |
| FL45C20 | 1.2% | 8.7% | 0.166 | 0.13 | [0.03, 0.60] | 0.006* |
| FL45C30 | 33.5% | 34.0% | 0.000 | 0.98 | [0.58, 1.65] | 1.000 |
| FL45C40 | 86.2% | 79.6% | 0.077 | 1.60 | [0.84, 3.07] | 0.208 |
| FL70C0 | 10.2% | 15.5% | 0.068 | 0.62 | [0.30, 1.28] | 0.265 |
| FL70C10 | 14.4% | 11.7% | 0.028 | 1.27 | [0.61, 2.67] | 0.649 |
| FL70C20 | 37.7% | 33.0% | 0.040 | 1.23 | [0.73, 2.06] | 0.513 |
| FL70C30 | 79.0% | 73.8% | 0.052 | 1.34 | [0.75, 2.38] | 0.396 |
| FL70C40 | 95.2% | 96.1% | 0.003 | 0.80 | [0.24, 2.74] | 0.962 |

Note. Values are percentages. φ = phi; OR = odds ratio. *P < 0.05.

**Supplementary Table 12.** Comparison of Surgical Recommendation Rates for Male Images by Evaluator Gender

| **Image** | **Female(N=167)** | **Male(N=103)** | **Φ** | **OR** | **95% CI** | **P value** |
| --- | --- | --- | --- | --- | --- | --- |
| ML30C0 | 91.0% | 90.3% | 0.000 | 1.09 | [0.47, 2.53] | 1.000 |
| ML30C10 | 88.0% | 81.6% | 0.079 | 1.66 | [0.84, 3.29] | 0.197 |
| ML30C20 | 47.3% | 50.5% | 0.023 | 0.88 | [0.54, 1.44] | 0.702 |
| ML30C30 | 64.7% | 60.2% | 0.037 | 1.21 | [0.73, 2.01] | 0.542 |
| ML30C40 | 67.7% | 71.8% | 0.036 | 0.82 | [0.48, 1.40] | 0.557 |
| ML45C0 | 47.9% | 44.7% | 0.024 | 1.14 | [0.70, 1.87] | 0.694 |
| ML45C10 | 11.4% | 8.7% | 0.030 | 1.34 | [0.58, 3.09] | 0.627 |
| ML45C20 | 5.4% | 9.7% | 0.067 | 0.53 | [0.21, 1.35] | 0.270 |
| ML45C30 | 55.1% | 54.4% | 0.000 | 1.03 | [0.63, 1.69] | 1.000 |
| ML45C40 | 77.8% | 81.6% | 0.035 | 0.79 | [0.43, 1.47] | 0.565 |
| ML70C0 | 9.0% | 9.7% | 0.000 | 0.92 | [0.40, 2.13] | 1.000 |
| ML70C10 | 17.4% | 12.6% | 0.053 | 1.45 | [0.72, 2.95] | 0.383 |
| ML70C20 | 35.3% | 35.9% | 0.000 | 0.97 | [0.58, 1.63] | 1.000 |
| ML70C30 | 69.5% | 68.0% | 0.008 | 1.07 | [0.63, 1.82] | 0.902 |
| ML70C40 | 89.8% | 93.2% | 0.044 | 0.64 | [0.26, 1.61] | 0.466 |

Note. Values are percentages. φ = phi; OR = odds ratio. *P < 0.05.
